# Supplementary material for: Stochastic and Deterministic Processes Regulate Phytoplankton Assemblages in a Temperate Coastal Ecosystem
Source: Microbiol Spectr. 2022 Oct 12;10(6):e02427-22. doi: 10.1128/spectrum.02427-22 (PMC9769578; doi:10.1128/spectrum.02427-22)
Supplement: Supplemental file 1 — Supplemental materials and methods, Tables S1 to S9, and Fig. S1 to S10. Download spectrum.02427-22-s0001.pdf, PDF file, 1.3 MB [file spectrum.02427-22-s0001.pdf]

Supplementary Information

Supplementary Materials and Methods

DNA barcoding

Different molecular barcodes of 10 bp were added to both forward and reverse primers to tag amplicons and allow to differentiate them after sequencing. Polymerase chain reaction (PCR) mixtures comprised 1 μL of DNA, 12.5 μL of DreamTaq Green PCR Master Mix (Thermo Fisher Scientific, U.S.A.), 1 μL of each primer (10 μmol L^{-1}), and 9.5 μL nuclease-free water in a total volume of 25 μL. PCR settings included an initial step of denaturation at 94 ^{o}C for 2 min, 25 cycles of denaturation at 94 ^{o}C for 15 s, annealing at 55 ^{o}C for 30 s, an extension at 72 ^{o}C for 1 min and 30 sec, and a final step of extension at 72 ^{o}C for 5 min. About 4 μL PCR product was used to check amplification on 1% agarose gel. The remaining amplicon products from five different PCR reactions of each sample were pooled and purified together using the QIAquick PCR purification kit (Qiagen, Germany), according to the manufacturer's instructions. DNA concentrations after purification were measured with a Qubit 2.0 fluorometer (Thermo Fischer Scientific Inc) with the dsDNA High Sensitivity Assay Kit (Life Technologies Corp., U.S.A.) and adjusted at equal concentrations depending on the sequencing run (20 to 47 ng/μL).

A diversity and statistical analysis

The alpha diversity indices Richness, Shannon and Simpson were calculated with the "vegan" package [1] and Faith's phylogenetic diversity index was calculated with the "Picante" package [2]. Heatmaps were generated to illustrate the overall phytoplankton community composition and diversity of the 30 most common genera in all samples (i.e, 0.5 % of reads in the entire dataset), using the "Ampvis2" R-package [3] after pooling ASVs belonging to the same genus. Monthly boxplots were built using the "ggplot2" package [4] to illustrate seasonal variations of the environmental variables, the alpha diversity indexes, the phylogenetic indexes and the different phytoplankton groups. To test how the environmental variables and the different phytoplankton groups' abundance, biomass and number of reads differed among stations the non-parametric test Kruskal-Wallis was performed, followed by a post hoc Nemenyi test (package "PMCMRplus") [5]. To explore the seasonal environmental gradient a Principal Component Analysis (PCA) was performed on environmental variables using the "ade4", "FactoMineR" and "factoextra" packages [6, 7, 8]. The significance of each environmental variable in driving the phytoplankton community structure was assessed by

distance-based redundancy analysis (db-RDA) [9] based on Bray-Curtis dissimilarity distance matrix using the "microeco" R package v.0.6.0 [10]. The significance of db-RDA models was tested with a permutation test using the *permutest* function with 999 permutations.

Community assembly

Mantel correlograms were applied to detect phylogenetic signals (closely related taxa have similar habitat associations). The phylogenetic signal was evaluated for PAR, temperature, salinity, nitrite and nitrate, phosphate, and silicate. It allowed characterizing the continuous correlations by comparing each matrix of between-ASV environmental optima (or "niche value") differences and the second matrix of between-ASV phylogenetic distances. For example, for PAR we took all the records of a given ASV (this was done for all ASVs) and recorded the PAR of each record, the ASV's abundance in each record, and then found the abundance -weighted mean of PAR. This is the ASV's 'niche value' for PAR. The analogous procedure was used to estimate ASV niche values for all the other environmental parameters. To summarize major trends in this relationship, between-ASVs niche differences were placed in phylogenetic distance bins and median niche difference was found in each bin (which are represented by the squares in the graph). X axis shows the phylogenetic distances (0: lowest, 1.0 maximum). Y axis shows the correlation between environmental optima and phylogenetic distances. All mantel correlograms showed positive correlation between niche optima and phylogenetic distances at short phylogenetic distances, which justifies the utilisation of the phylogenetic metrics to infer community assembly [11].

The βNRI was calculated as the difference between the observed βMPD and the mean of the βMPD null models divided by the standard deviation of the null models. Beta net relatedness values (βNRI) lower than expected (i.e., βNRI < -2) indicate a dominance of homogeneous selection. In contrast, βNRI values which are greater than expected (i.e., βNRI > 2) indicate that communities are experiencing heterogeneous selection. When the deviation was low (i.e., -2 < βNRI ≤ 2), an additional step was conducted to define whether the beta diversity of the communities could be structured by dispersal or drift (Table 1). In this step, the Raup–Crick metric (RCbray) [12] was calculated using the Jaccard's distance. For this, RCbray compares the measured b-diversity against the b-diversity that would be obtained if drift was driving community turnover (i.e., under random community assembly). The randomization was run 999 times based on the presence-absence of all ASVs across each pairwise community comparison that is randomized. RCbray values less than - 0.95 indicate that community turnover is driven by dispersal limitation, RCbray values greater than 0.95 indicate

homogeneous dispersal respectively, and values between -0.95 and +0.95 point to a community assembly governed by drift or other undominated mechanisms.

References

1. Oksanen J, Blanchet FG, Friendly M, Kindt R, Legendre P, McGlinn D, et al. vegan: Community Ecology Package. 2020.
2. Kembel SW, Cowan PD, Helmus MR, Cornwell WK, Morlon H, Ackerly DD, et al. Picante: R tools for integrating phylogenies and ecology. *Bioinformatics* 2010; **26**: 1463–1464.
3. Andersen KS, Kirkegaard RH, Karst SM, Albertsen M. ampvis2: an R package to analyse and visualise 16S rRNA amplicon data. *bioRxiv* 2018.
4. Wickham H. ggplot2. 2016. Springer International Publishing, Cham.
5. Pohlert T. PMCMR: calculate pairwise multiple comparisons of mean rank sums. *R package version* 2015; **1**.
6. Dray S, Dufour AB, Chessel D. The ade4 Package — II: Two-table and K-table Methods. 2007; **7**: 6.
7. Lê S, Josse J, Husson F. FactoMineR: An R Package for Multivariate Analysis. *Journal of Statistical Software* 2008; **25**: 1–18.
8. Kassambara A, Mundt F. factoextra: Extract and Visualize the Results of Multivariate Data Analyses. 2020.
9. Legendre P, Anderson MJ. DISTANCE-BASED REDUNDANCY ANALYSIS: TESTING MULTISPECIES RESPONSES IN MULTIFACTORIAL ECOLOGICAL EXPERIMENTS. *Ecological Monographs* 1999; **69**: 24.
10. Liu C, Cui Y, Li X, Yao M. *microeco* : an R package for data mining in microbial community ecology. *FEMS Microbiology Ecology* 2021; **97**: fiaa255.

11. Stegen JC, Lin X, Konopka AE, Fredrickson J. Stochastic and deterministic assembly processes in subsurface microbial communities. *The ISME Journal* 2012; 12.

12. Chase JM, Kraft NJB, Smith KG, Vellend M, Inouye BD. Using null models to disentangle variation in community dissimilarity from variation in α-diversity. *Ecosphere* 2011; **2**: art24.

Supplementary Tables

Table S1. List of the environmental variables Photosynthetic Active Radiation (PAR_{10m}, E m^{-2} d^{-1}), sea surface temperature (SST, °C), m^{-1}), salinity (SSU), nitrites (NO_2, µM), nitrates (NO_3, µM), phosphates (PO_4, µM), silicates (SiO_4, µM), chlorophyll-a (Chla, µg L^{-1}), wind speed (m s^{-1}) wind stress (Pa) and Rainfall (Kg m^2) in the SOMLIT (S1, S2) and DYPHYRAD (R1, R2, R4) stations in the coastal waters of the Eastern English Channel.

ID	Stations	Year	Month	Date	Longlitude	Latitude	Abiotic parameters									
S1.20160307	S1	2016	3	07/03/2016	1.521	50.688	PAR	Temp	S	NO2	NO3	PO4	Si(OH)4	Chla	WindStress	Rainfall
S1.20160322	S1	2016	3	22/03/2016	1.521	50.688	PAR	Temp	S	NO2	NO3	PO4	Si(OH)4	Chla	WindStress	Rainfall
S2.20160322	S2	2016	3	22/03/2016	1.417	50.688	PAR	Temp	S	NO2	NO3	PO4	Si(OH)4	Chla	WindStress	Rainfall
S1.20160405	S1	2016	4	05/04/2016	1.521	50.688	PAR	Temp	S	NO2	NO3	PO4	Si(OH)4	Chla	WindStress	Rainfall
S2.20160405	S2	2016	4	05/04/2016	1.417	50.688	PAR	Temp	S	NO2	NO3	PO4	Si(OH)4	Chla	WindStress	Rainfall
S1.20160420	S1	2016	4	20/04/2016	1.521	50.688	PAR	Temp	S	NO2	NO3	PO4	Si(OH)4	Chla	WindStress	Rainfall
S1.20160509	S1	2016	5	09/05/2016	1.521	50.688	PAR	Temp	S	NO2	NO3	PO4	Si(OH)4	Chla	WindStress	Rainfall
S2.20160509	S2	2016	5	09/05/2016	1.417	50.688	PAR	Temp	S	NO2	NO3	PO4	Si(OH)4	Chla	WindStress	Rainfall
S1.20160523	S1	2016	5	23/05/2016	1.521	50.688	PAR	Temp	S	NO2	NO3	PO4	Si(OH)4	Chla	WindStress	Rainfall
S2.20160523	S2	2016	5	23/05/2016	1.417	50.688	PAR	Temp	S	NO2	NO3	PO4	Si(OH)4	Chla	WindStress	Rainfall
S1.20160606	S1	2016	6	06/06/2016	1.521	50.688	PAR	Temp	S	NO2	NO3	PO4	Si(OH)4	Chla	WindStress	Rainfall
S1.20160621	S1	2016	6	21/06/2016	1.521	50.688	PAR	Temp	S	NO2	NO3	PO4	Si(OH)4	Chla	WindStress	Rainfall
S1.20160706	S1	2016	7	06/07/2016	1.521	50.688	PAR	Temp	S	NO2	NO3	PO4	Si(OH)4	Chla	WindStress	Rainfall
S1.20160721	S1	2016	7	21/07/2016	1.521	50.688	PAR	Temp	S	NO2	NO3	PO4	Si(OH)4	Chla	WindStress	Rainfall
S1.20160902	S1	2016	9	02/09/2016	1.521	50.688	PAR	Temp	S	NO2	NO3	PO4	Si(OH)4	Chla	WindStress	Rainfall
S1.20160919	S1	2016	9	19/09/2016	1.521	50.688	PAR	Temp	S	NO2	NO3	PO4	Si(OH)4	Chla	WindStress	Rainfall
S2.20160919	S2	2016	9	19/09/2016	1.417	50.688	PAR	Temp	S	NO2	NO3	PO4	Si(OH)4	Chla	WindStress	Rainfall
S1.20161003	S1	2016	10	03/10/2016	1.521	50.688	PAR	Temp	S	NO2	NO3	PO4	Si(OH)4	Chla	WindStress	Rainfall
S2.20161003	S2	2016	10	03/10/2016	1.417	50.688	PAR	Temp	S	NO2	NO3	PO4	Si(OH)4	Chla	WindStress	Rainfall
S1.20161103	S1	2016	11	03/11/2016	1.521	50.688	PAR	Temp	S	NO2	NO3	PO4	Si(OH)4	Chla	WindStress	Rainfall
S2.20161103	S2	2016	11	03/11/2016	1.417	50.688	PAR	Temp	S	NO2	NO3	PO4	Si(OH)4	Chla	WindStress	Rainfall
S1.20161114	S1	2016	11	14/11/2016	1.521	50.688	PAR	Temp	S	NO2	NO3	PO4	Si(OH)4		WindStress	Rainfall
S2.20161114	S2	2016	11	14/11/2016	1.417	50.688	PAR	Temp	S	NO2	NO3	PO4	Si(OH)4		WindStress	Rainfall
S1.20161201	S1	2016	12	01/12/2016	1.521	50.688	PAR	Temp	S	NO2	NO3	PO4	Si(OH)4	Chla	WindStress	Rainfall
S2.20161201	S2	2016	12	01/12/2016	1.417	50.688	PAR	Temp	S	NO2	NO3	PO4	Si(OH)4	Chla	WindStress	Rainfall
S1.20161212	S1	2016	12	12/12/2016	1.521	50.688	PAR	Temp	S	NO2	NO3	PO4	Si(OH)4	Chla	WindStress	Rainfall
S2.20161212	S2	2016	12	12/12/2016	1.417	50.688	PAR	Temp	S	NO2	NO3	PO4	Si(OH)4	Chla	WindStress	Rainfall
S1.20170130	S1	2017	1	30/01/2017	1.521	50.688	PAR	Temp	S	NO2	NO3	PO4	Si(OH)4	Chla	WindStress	Rainfall

S2.20170130	S2	2017	1	30/01/2017	1.417	50.688	PAR	Temp	S	NO2	NO3	PO4	Si(OH)4	Chla	WindStress	Rainfall
S1.20170214	S1	2017	2	14/02/2017	1.521	50.688	PAR	Temp	S	NO2	NO3	PO4	Si(OH)4	Chla	WindStress	Rainfall
S1.20170313	S1	2017	3	13/03/2017	1.521	50.688	PAR	Temp	S	NO2	NO3	PO4	Si(OH)4	Chla	WindStress	Rainfall
S2.20170313	S2	2017	3	13/03/2017	1.417	50.688	PAR	Temp	S	NO2	NO3	PO4	Si(OH)4	Chla	WindStress	Rainfall
S1.20170327	S1	2017	3	27/03/2017	1.521	50.688	PAR	Temp	S	NO2	NO3	PO4	Si(OH)4	Chla	WindStress	Rainfall
S2.20170327	S2	2017	3	27/03/2017	1.417	50.688	PAR	Temp	S	NO2	NO3	PO4	Si(OH)4	Chla	WindStress	Rainfall
S1.20170411	S1	2017	4	11/04/2017	1.521	50.688	PAR	Temp	S	NO2	NO3	PO4	Si(OH)4	Chla	WindStress	Rainfall
S2.20170411	S2	2017	4	11/04/2017	1.417	50.688	PAR	Temp	S	NO2	NO3	PO4	Si(OH)4	Chla	WindStress	Rainfall
S1.20170510	S1	2017	5	10/05/2017	1.521	50.688	PAR	Temp	S	NO2	NO3	PO4	Si(OH)4	Chla	WindStress	Rainfall
S2.20170510	S2	2017	5	10/05/2017	1.417	50.688	PAR	Temp	S	NO2	NO3	PO4	Si(OH)4	Chla	WindStress	Rainfall
S1.20170524	S1	2017	5	24/05/2017	1.521	50.688	PAR	Temp	S	NO2	NO3	PO4	Si(OH)4	Chla	WindStress	Rainfall
S2.20170524	S2	2017	5	24/05/2017	1.417	50.688	PAR	Temp	S	NO2	NO3	PO4	Si(OH)4	Chla	WindStress	Rainfall
S1.20170627	S1	2017	6	27/06/2017	1.521	50.688	PAR	Temp	S	NO2		PO4	Si(OH)4	Chla	WindStress	Rainfall
S2.20170627	S2	2017	6	27/06/2017	1.417	50.688	PAR	Temp	S	NO2		PO4	Si(OH)4	Chla	WindStress	Rainfall
S1.20170711	S1	2017	7	11/07/2017	1.521	50.688	PAR	Temp	S	NO2		PO4	Si(OH)4	Chla	WindStress	Rainfall
S1.20170724	S1	2017	7	24/07/2017	1.521	50.688	PAR			NO2		PO4	Si(OH)4	Chla	WindStress	Rainfall
S2.20170724	S2	2017	7	24/07/2017	1.417	50.688	PAR			NO2		PO4	Si(OH)4	Chla	WindStress	Rainfall
S1.20170907	S1	2017	9	07/09/2017	1.521	50.688	PAR	Temp	S	NO2	NO3	PO4	Si(OH)4	Chla	WindStress	Rainfall
S2.20170907	S2	2017	9	07/09/2017	1.417	50.688	PAR	Temp	S	NO2	NO3	PO4	Si(OH)4	Chla	WindStress	Rainfall
S1.20170918	S1	2017	9	18/09/2017	1.521	50.688	PAR	Temp	S	NO2	NO3	PO4	Si(OH)4	Chla	WindStress	Rainfall
S2.20170918	S2	2017	9	18/09/2017	1.417	50.688	PAR	Temp	S	NO2	NO3	PO4	Si(OH)4	Chla	WindStress	Rainfall
S1.20171004	S1	2017	10	04/10/2017	1.521	50.688	PAR	Temp	S	NO2	NO3	PO4	Si(OH)4	Chla	WindStress	Rainfall
S1.20171018	S1	2017	10	18/10/2017	1.521	50.688	PAR	Temp	S	NO2	NO3	PO4	Si(OH)4	Chla	WindStress	Rainfall
S2.20171018	S2	2017	10	18/10/2017	1.417	50.688	PAR	Temp	S	NO2	NO3	PO4	Si(OH)4	Chla	WindStress	Rainfall
S1.20171102	S1	2017	11	02/11/2017	1.521	50.688	PAR	Temp	S	NO2	NO3	PO4	Si(OH)4	Chla	WindStress	Rainfall
S1.20171116	S1	2017	11	16/11/2017	1.521	50.688	PAR	Temp	S	NO2	NO3	PO4	Si(OH)4	Chla	WindStress	Rainfall
S2.20171116	S2	2017	11	16/11/2017	1.417	50.688	PAR	Temp	S	NO2	NO3	PO4	Si(OH)4	Chla	WindStress	Rainfall
S1.20171219	S1	2017	12	19/12/2017	1.521	50.688	PAR	Temp	S	NO2	NO3	PO4	Si(OH)4	Chla	WindStress	Rainfall
S2.20171219	S2	2017	12	19/12/2017	1.417	50.688	PAR	Temp	S	NO2	NO3	PO4	Si(OH)4	Chla	WindStress	Rainfall
R1.20180207	R1	2018	2	07/02/2018	1.567	50.799	PAR	Temp	S	NO2	NO3	PO4	Si(OH)4	Chla	WindStress	Rainfall
R2.20180207	R2	2018	2	07/02/2018	1.542	50.799	PAR	Temp	S	NO2	NO3	PO4	Si(OH)4	Chla	WindStress	Rainfall
R4.20180207	R4	2018	2	07/02/2018	1.452	50.799	PAR	Temp	S	NO2	NO3	PO4	Si(OH)4	Chla	WindStress	Rainfall
S1.20180216	S1	2018	2	16/02/2018	1.521	50.688	PAR	Temp	S	NO2	NO3	PO4	Si(OH)4	Chla	WindStress	Rainfall
S2.20180216	S2	2018	2	16/02/2018	1.417	50.688	PAR	Temp	S	NO2	NO3	PO4	Si(OH)4	Chla	WindStress	Rainfall
R1.20180228	R1	2018	2	28/02/2018	1.567	50.799	PAR	Temp	S	NO2	NO3	PO4	Si(OH)4	Chla	WindStress	Rainfall
R2.20180228	R2	2018	2	28/02/2018	1.542	50.799	PAR	Temp	S	NO2	NO3	PO4	Si(OH)4	Chla	WindStress	Rainfall
R4.20180228	R4	2018	2	28/02/2018	1.452	50.799	PAR	Temp	S	NO2	NO3	PO4	Si(OH)4	Chla	WindStress	Rainfall
S1.20180302	S1	2018	3	02/03/2018	1.521	50.688	PAR	Temp	S	NO2	NO3	PO4	Si(OH)4	Chla	WindStress	Rainfall
S2.20180302	S2	2018	3	02/03/2018	1.417	50.688	PAR	Temp	S	NO2	NO3	PO4	Si(OH)4	Chla	WindStress	Rainfall
R1.20180315	R1	2018	3	15/03/2018	1.567	50.799	PAR	Temp	S	NO2	NO3	PO4	Si(OH)4	Chla	WindStress	Rainfall
R2.20180315	R2	2018	3	15/03/2018	1.542	50.799	PAR	Temp	S	NO2	NO3	PO4	Si(OH)4	Chla	WindStress	Rainfall
R4.20180315	R4	2018	3	15/03/2018	1.452	50.799	PAR	Temp	S	NO2	NO3	PO4	Si(OH)4	Chla	WindStress	Rainfall
S1.20180319	S1	2018	3	19/03/2018	1.521	50.688	PAR	Temp	S	NO2	NO3	PO4	Si(OH)4	Chla	WindStress	Rainfall
R1.20180322	R1	2018	3	22/03/2018	1.567	50.799	PAR	Temp	S	NO2	NO3	PO4	Si(OH)4	Chla	WindStress	Rainfall
R1.20180329	R1	2018	3	29/03/2018	1.567	50.799	PAR	Temp	S	NO2	NO3	PO4	Si(OH)4	Chla	WindStress	Rainfall
R2.20180329	R2	2018	3	29/03/2018	1.542	50.799	PAR	Temp	S	NO2	NO3	PO4	Si(OH)4	Chla	WindStress	Rainfall
R1.20180411	R1	2018	4	11/04/2018	1.567	50.799	PAR	Temp	S	NO2	NO3	PO4	Si(OH)4	Chla	WindStress	Rainfall
R2.20180411	R2	2018	4	11/04/2018	1.542	50.799	PAR	Temp	S	NO2	NO3	PO4	Si(OH)4	Chla	WindStress	Rainfall

R4.20180411	R4	2018	4	11/04/2018	1.452	50.799	PAR	Temp	S	NO2	NO3	PO4	Si(OH)4	Chla	WindStress	Rainfall
S1.20180416	S1	2018	4	16/04/2018	1.521	50.688	PAR	Temp	S	NO2	NO3	PO4	Si(OH)4	Chla	WindStress	Rainfall
S2.20180416	S2	2018	4	16/04/2018	1.417	50.688	PAR	Temp	S	NO2	NO3	PO4	Si(OH)4	Chla	WindStress	Rainfall
R1.20180417	R1	2018	4	17/04/2018	1.567	50.799	PAR	Temp	S	NO2	NO3	PO4	Si(OH)4	Chla	WindStress	Rainfall
R2.20180417	R2	2018	4	17/04/2018	1.542	50.799	PAR	Temp	S	NO2	NO3	PO4	Si(OH)4	Chla	WindStress	Rainfall
R4.20180417	R4	2018	4	17/04/2018	1.452	50.799	PAR	Temp	S	NO2	NO3	PO4	Si(OH)4	Chla	WindStress	Rainfall
S1.20180503	S1	2018	5	03/05/2018	1.521	50.688	PAR	Temp	S	NO2	NO3	PO4	Si(OH)4	Chla	WindStress	Rainfall
R1.20180504	R1	2018	5	04/05/2018	1.567	50.799	PAR			NO2	NO3	PO4	Si(OH)4	Chla	WindStress	Rainfall
R2.20180504	R2	2018	5	04/05/2018	1.542	50.799	PAR			NO2	NO3	PO4	Si(OH)4	Chla	WindStress	Rainfall
R4.20180504	R4	2018	5	04/05/2018	1.452	50.799	PAR			NO2	NO3	PO4	Si(OH)4	Chla	WindStress	Rainfall
R1.20180511	R1	2018	5	11/05/2018	1.567	50.799	PAR	Temp	S	NO2	NO3	PO4	Si(OH)4	Chla	WindStress	Rainfall
R1.20180516	R1	2018	5	16/05/2018	1.567	50.799	PAR	Temp	S	NO2	NO3	PO4	Si(OH)4	Chla	WindStress	Rainfall
R1.20180523	R1	2018	5	23/05/2018	1.567	50.799	PAR	Temp	S	NO2	NO3	PO4	Si(OH)4	Chla	WindStress	Rainfall
S1.20180529	S1	2018	5	29/05/2018	1.521	50.688	PAR	Temp	S	NO2	NO3	PO4	Si(OH)4	Chla	WindStress	Rainfall
S2.20180529	S2	2018	5	29/05/2018	1.417	50.688	PAR	Temp	S	NO2	NO3	PO4	Si(OH)4	Chla	WindStress	Rainfall
R1.20180531	R1	2018	5	31/05/2018	1.567	50.799	PAR	Temp	S	NO2	NO3	PO4	Si(OH)4	Chla	WindStress	Rainfall
R2.20180531	R2	2018	5	31/05/2018	1.542	50.799	PAR	Temp	S	NO2	NO3	PO4	Si(OH)4	Chla	WindStress	Rainfall
R4.20180531	R4	2018	5	31/05/2018	1.452	50.799	PAR	Temp	S	NO2	NO3	PO4	Si(OH)4	Chla	WindStress	Rainfall
R1.20180605	R1	2018	6	05/06/2018	1.567	50.799	PAR	Temp	S	NO2	NO3	PO4	Si(OH)4	Chla	WindStress	Rainfall
R1.20180606	R1	2018	6	06/06/2018	1.567	50.799	PAR	Temp	S	NO2	NO3	PO4	Si(OH)4	Chla	WindStress	Rainfall
R1.20180607	R1	2018	6	07/06/2018	1.567	50.799	PAR	Temp	S	NO2	NO3	PO4	Si(OH)4	Chla	WindStress	Rainfall
R2.20180607	R2	2018	6	07/06/2018	1.542	50.799	PAR	Temp	S	NO2	NO3	PO4	Si(OH)4	Chla	WindStress	Rainfall
R4.20180607	R4	2018	6	07/06/2018	1.452	50.799	PAR	Temp	S	NO2	NO3	PO4	Si(OH)4	Chla	WindStress	Rainfall
R1.20180608	R1	2018	6	08/06/2018	1.567	50.799	PAR	Temp	S	NO2	NO3	PO4	Si(OH)4	Chla	WindStress	Rainfall
S1.20180612	S1	2018	6	12/06/2018	1.521	50.688	PAR	Temp	S	NO2	NO3	PO4	Si(OH)4	Chla	WindStress	Rainfall
R1.20180613	R1	2018	6	13/06/2018	1.567	50.799	PAR	Temp	S	NO2	NO3	PO4	Si(OH)4	Chla	WindStress	Rainfall
R2.20180613	R2	2018	6	13/06/2018	1.542	50.799	PAR	Temp	S	NO2	NO3	PO4	Si(OH)4	Chla	WindStress	Rainfall
R4.20180613	R4	2018	6	13/06/2018	1.452	50.799	PAR	Temp	S	NO2	NO3	PO4	Si(OH)4	Chla	WindStress	Rainfall
R1.20180618	R1	2018	6	18/06/2018	1.567	50.799	PAR	Temp	S	NO2	NO3	PO4	Si(OH)4	Chla	WindStress	Rainfall
R1.20180620	R1	2018	6	20/06/2018	1.567	50.799	PAR	Temp	S	NO2	NO3	PO4	Si(OH)4	Chla	WindStress	Rainfall
R4.20180620	R4	2018	6	20/06/2018	1.452	50.799	PAR	Temp	S	NO2	NO3	PO4	Si(OH)4	Chla	WindStress	Rainfall
R1.20180622	R1	2018	6	22/06/2018	1.567	50.799	PAR	Temp	S	NO2	NO3	PO4	Si(OH)4	Chla	WindStress	Rainfall
R2.20180622	R2	2018	6	22/06/2018	1.542	50.799	PAR	Temp	S	NO2	NO3	PO4	Si(OH)4	Chla	WindStress	Rainfall
R4.20180622	R4	2018	6	22/06/2018	1.452	50.799	PAR	Temp	S	NO2	NO3	PO4	Si(OH)4	Chla	WindStress	Rainfall
R1.20180625	R1	2018	6	25/06/2018	1.567	50.799	PAR	Temp	S	NO2	NO3	PO4	Si(OH)4	Chla	WindStress	Rainfall
R1.20180626	R1	2018	6	26/06/2018	1.567	50.799	PAR	Temp	S	NO2	NO3	PO4	Si(OH)4	Chla	WindStress	Rainfall
R1.20180627	R1	2018	6	27/06/2018	1.567	50.799	PAR	Temp	S	NO2	NO3	PO4	Si(OH)4	Chla	WindStress	Rainfall
R2.20180627	R2	2018	6	27/06/2018	1.542	50.799	PAR	Temp	S	NO2	NO3	PO4	Si(OH)4	Chla	WindStress	Rainfall
R4.20180627	R4	2018	6	27/06/2018	1.452	50.799	PAR	Temp	S	NO2	NO3	PO4	Si(OH)4	Chla	WindStress	Rainfall
R1.20180628	R1	2018	6	28/06/2018	1.567	50.799	PAR	Temp	S	NO2	NO3	PO4	Si(OH)4	Chla	WindStress	Rainfall
R4.20180628	R4	2018	6	28/06/2018	1.452	50.799	PAR	Temp	S	NO2	NO3	PO4	Si(OH)4	Chla	WindStress	Rainfall
S1.20180628	S1	2018	6	28/06/2018	1.521	50.688	PAR	Temp	S	NO2	NO3	PO4	Si(OH)4	Chla	WindStress	Rainfall
R1.20180629	R1	2018	6	29/06/2018	1.567	50.799	PAR	Temp	S	NO2	NO3	PO4	Si(OH)4	Chla	WindStress	Rainfall
R1.20180706	R1	2018	7	06/07/2018	1.567	50.799	PAR	Temp	S	NO2	NO3	PO4	Si(OH)4	Chla	WindStress	Rainfall
R4.20180706	R4	2018	7	06/07/2018	1.452	50.799	PAR	Temp	S	NO2	NO3	PO4	Si(OH)4	Chla	WindStress	Rainfall
R1.20180711	R1	2018	7	11/07/2018	1.567	50.799	PAR	Temp	S	NO2	NO3	PO4	Si(OH)4	Chla	WindStress	Rainfall
S1.20180712	S1	2018	7	12/07/2018	1.521	50.688	PAR	Temp	S	NO2	NO3	PO4	Si(OH)4	Chla	WindStress	Rainfall
S2.20180712	S2	2018	7	12/07/2018	1.417	50.688	PAR	Temp	S	NO2	NO3	PO4	Si(OH)4	Chla	WindStress	Rainfall

S1.20180727	S1	2018	7	27/07/2018	1.521	50.688	PAR	Temp	S	NO2	NO3	PO4	Si(OH)4	Chla	WindStress	Rainfall
S2.20180727	S2	2018	7	27/07/2018	1.417	50.688	PAR	Temp	S	NO2	NO3	PO4	Si(OH)4	Chla	WindStress	Rainfall
R1.20180828	R1	2018	8	28/08/2018	1.567	50.799	PAR	Temp	S	NO2	NO3	PO4	Si(OH)4	Chla	WindStress	Rainfall
R2.20180828	R2	2018	8	28/08/2018	1.542	50.799	PAR	Temp	S	NO2	NO3	PO4	Si(OH)4	Chla	WindStress	Rainfall
R4.20180828	R4	2018	8	28/08/2018	1.452	50.799	PAR	Temp	S	NO2	NO3	PO4	Si(OH)4	Chla	WindStress	Rainfall
R1.20180925	R1	2018	9	25/09/2018	1.567	50.799	PAR	Temp	S	NO2	NO3	PO4	Si(OH)4	Chla	WindStress	Rainfall
R2.20180925	R2	2018	9	25/09/2018	1.542	50.799	PAR	Temp	S	NO2	NO3	PO4	Si(OH)4	Chla	WindStress	Rainfall
R4.20180925	R4	2018	9	25/09/2018	1.452	50.799	PAR	Temp	S	NO2	NO3	PO4	Si(OH)4	Chla	WindStress	Rainfall
S1.20180927	S1	2018	9	27/09/2018	1.521	50.688	PAR	Temp	S	NO2	NO3	PO4	Si(OH)4	Chla	WindStress	Rainfall
S2.20180927	S2	2018	9	27/09/2018	1.417	50.688	PAR	Temp	S	NO2	NO3	PO4	Si(OH)4	Chla	WindStress	Rainfall
S1.20181008	S1	2018	10	08/10/2018	1.521	50.688	PAR	Temp	S	NO2	NO3	PO4	Si(OH)4	Chla	WindStress	Rainfall
S2.20181008	S2	2018	10	08/10/2018	1.417	50.688	PAR	Temp	S	NO2	NO3	PO4	Si(OH)4	Chla	WindStress	Rainfall
R1.20181011	R1	2018	10	11/10/2018	1.567	50.799	PAR	Temp	S	NO2	NO3	PO4	Si(OH)4	Chla	WindStress	Rainfall
R1.20181019	R1	2018	10	19/10/2018	1.567	50.799	PAR	Temp	S	NO2	NO3	PO4	Si(OH)4	Chla	WindStress	Rainfall
R2.20181019	R2	2018	10	19/10/2018	1.542	50.799	PAR	Temp	S	NO2	NO3	PO4	Si(OH)4	Chla	WindStress	Rainfall
R4.20181019	R4	2018	10	19/10/2018	1.452	50.799	PAR	Temp	S	NO2	NO3	PO4	Si(OH)4	Chla	WindStress	Rainfall
S1.20181024	S1	2018	10	24/10/2018	1.521	50.688	PAR	Temp	S	NO2	NO3	PO4	Si(OH)4	Chla	WindStress	Rainfall
S2.20181024	S2	2018	10	24/10/2018	1.417	50.688	PAR	Temp	S	NO2	NO3	PO4	Si(OH)4	Chla	WindStress	Rainfall
R1.20181025	R1	2018	10	25/10/2018	1.567	50.799	PAR	Temp	S	NO2	NO3	PO4	Si(OH)4	Chla	WindStress	Rainfall
R2.20181025	R2	2018	10	25/10/2018	1.542	50.799	PAR	Temp	S	NO2	NO3	PO4	Si(OH)4	Chla	WindStress	Rainfall
R4.20181025	R4	2018	10	25/10/2018	1.452	50.799	PAR	Temp	S	NO2	NO3	PO4	Si(OH)4	Chla	WindStress	Rainfall
R1.20181114	R1	2018	11	14/11/2018	1.567	50.799	PAR	Temp	S	NO2	NO3	PO4	Si(OH)4	Chla	WindStress	Rainfall
R1.20181121	R1	2018	11	21/11/2018	1.567	50.799	PAR	Temp	S	NO2	NO3	PO4	Si(OH)4	Chla	WindStress	Rainfall
R2.20181121	R2	2018	11	21/11/2018	1.542	50.799	PAR	Temp	S	NO2	NO3	PO4	Si(OH)4	Chla	WindStress	Rainfall
S1.20181122	S1	2018	11	22/11/2018	1.521	50.688	PAR	Temp	S	NO2	NO3	PO4	Si(OH)4	Chla	WindStress	Rainfall
S2.20181122	S2	2018	11	22/11/2018	1.417	50.688	PAR	Temp	S	NO2	NO3	PO4	Si(OH)4	Chla	WindStress	Rainfall
S1.20181205	S1	2018	12	05/12/2018	1.521	50.688	PAR	Temp	S	NO2	NO3	PO4	Si(OH)4	Chla	WindStress	Rainfall
R1.20181206	R1	2018	12	06/12/2018	1.567	50.799	PAR	Temp	S	NO2	NO3	PO4	Si(OH)4	Chla	WindStress	Rainfall
R2.20181206	R2	2018	12	06/12/2018	1.542	50.799	PAR	Temp	S	NO2	NO3	PO4	Si(OH)4	Chla	WindStress	Rainfall
R1.20181213	R1	2018	12	13/12/2018	1.567	50.799	PAR	Temp	S	NO2	NO3	PO4	Si(OH)4	Chla	WindStress	Rainfall
R2.20181213	R2	2018	12	13/12/2018	1.542	50.799	PAR	Temp	S	NO2	NO3	PO4	Si(OH)4	Chla	WindStress	Rainfall
R4.20181213	R4	2018	12	13/12/2018	1.452	50.799	PAR	Temp	S	NO2	NO3	PO4	Si(OH)4	Chla	WindStress	Rainfall
R1.20190118	R1	2019	1	18/01/2019	1.567	50.799	PAR	Temp	S	NO2	NO3	PO4	Si(OH)4	Chla	WindStress	Rainfall
R2.20190118	R2	2019	1	18/01/2019	1.542	50.799	PAR	Temp	S		NO3	PO4	Si(OH)4	Chla	WindStress	Rainfall
R1.20190124	R1	2019	1	24/01/2019	1.567	50.799	PAR	Temp	S					Chla	WindStress	Rainfall
R2.20190124	R2	2019	1	24/01/2019	1.542	50.799	PAR	Temp	S					Chla	WindStress	Rainfall
R4.20190124	R4	2019	1	24/01/2019	1.452	50.799	PAR	Temp	S					Chla	WindStress	Rainfall
S1.20190219	S1	2019	2	19/02/2019	1.521	50.688	PAR	Temp	S	NO2	NO3	PO4	Si(OH)4	Chla	WindStress	Rainfall
S2.20190219	S2	2019	2	19/02/2019	1.417	50.688	PAR	Temp	S	NO2	NO3	PO4	Si(OH)4	Chla	WindStress	Rainfall
R1.20190222	R1	2019	2	22/02/2019	1.567	50.799	PAR	Temp	S	NO2	NO3	PO4	Si(OH)4	Chla	WindStress	Rainfall
R2.20190222	R2	2019	2	22/02/2019	1.542	50.799	PAR	Temp	S	NO2	NO3	PO4	Si(OH)4	Chla	WindStress	Rainfall
R4.20190222	R4	2019	2	22/02/2019	1.452	50.799	PAR	Temp	S	NO2	NO3	PO4	Si(OH)4	Chla	WindStress	Rainfall
S1.20190319	S1	2019	3	19/03/2019	1.521	50.688	PAR	Temp	S	NO2	NO3	PO4	Si(OH)4	Chla	WindStress	Rainfall
S2.20190319	S2	2019	3	19/03/2019	1.417	50.688	PAR	Temp	S	NO2	NO3	PO4	Si(OH)4	Chla	WindStress	Rainfall
R1.20190322	R1	2019	3	22/03/2019	1.567	50.799	PAR	Temp	S	NO2	NO3	PO4	Si(OH)4	Chla	WindStress	Rainfall
R2.20190322	R2	2019	3	22/03/2019	1.542	50.799	PAR	Temp	S	NO2	NO3	PO4	Si(OH)4	Chla	WindStress	Rainfall
R4.20190322	R4	2019	3	22/03/2019	1.452	50.799	PAR	Temp	S	NO2	NO3	PO4	Si(OH)4	Chla	WindStress	Rainfall
R1.20190327	R1	2019	3	27/03/2019	1.567	50.799	PAR	Temp	S	NO2	NO3	PO4	Si(OH)4	Chla	WindStress	Rainfall

R2.20190327	R2	2019	3	27/03/2019	1.542	50.799	PAR	Temp	S	NO2	NO3	PO4	Si(OH)4	Chla	WindStress	Rainfall
R4.20190327	R4	2019	3	27/03/2019	1.452	50.799	PAR	Temp	S	NO2	NO3	PO4	Si(OH)4	Chla	WindStress	Rainfall
S1.20190404	S1	2019	4	04/04/2019	1.521	50.688	PAR	Temp	S	NO2	NO3	PO4	Si(OH)4	Chla	WindStress	Rainfall
R1.20190405	R1	2019	4	05/04/2019	1.567	50.799	PAR	Temp	S	NO2	NO3	PO4	Si(OH)4	Chla	WindStress	Rainfall
R1.20190411	R1	2019	4	11/04/2019	1.567	50.799	PAR	Temp	S	NO2	NO3	PO4	Si(OH)4	Chla	WindStress	Rainfall
R2.20190411	R2	2019	4	11/04/2019	1.542	50.799	PAR	Temp	S	NO2	NO3	PO4	Si(OH)4	Chla	WindStress	Rainfall
R1.20190416	R1	2019	4	16/04/2019	1.567	50.799	PAR	Temp	S	NO2	NO3	PO4	Si(OH)4	Chla	WindStress	Rainfall
R2.20190416	R2	2019	4	16/04/2019	1.542	50.799	PAR	Temp	S	NO2	NO3	PO4	Si(OH)4	Chla	WindStress	Rainfall
R4.20190416	R4	2019	4	16/04/2019	1.452	50.799	PAR	Temp	S	NO2	NO3	PO4	Si(OH)4	Chla	WindStress	Rainfall
S1.20190417	S1	2019	4	17/04/2019	1.521	50.688	PAR	Temp	S	NO2	NO3	PO4	Si(OH)4	Chla	WindStress	Rainfall
S2.20190417	S2	2019	4	17/04/2019	1.417	50.688	PAR	Temp	S	NO2	NO3	PO4	Si(OH)4	Chla	WindStress	Rainfall
R1.20190426	R1	2019	4	26/04/2019	1.567	50.799	PAR	Temp	S	NO2	NO3	PO4	Si(OH)4	Chla	WindStress	Rainfall
S1.20190516	S1	2019	5	16/05/2019	1.521	50.688	PAR	Temp		NO2	NO3	PO4	Si(OH)4	Chla	WindStress	Rainfall
S2.20190516	S2	2019	5	16/05/2019	1.417	50.688	PAR	Temp		NO2	NO3	PO4	Si(OH)4	Chla	WindStress	Rainfall
S1.20190603	S1	2019	6	03/06/2019	1.521	50.688	PAR	Temp	S	NO2	NO3	PO4	Si(OH)4	Chla	WindStress	Rainfall
R1.20190605	R1	2019	6	05/06/2019	1.567	50.799	PAR	Temp	S	NO2	NO3	PO4	Si(OH)4	Chla	WindStress	Rainfall
R1.20190607	R1	2019	6	07/06/2019	1.567	50.799	PAR	Temp	S	NO2	NO3	PO4	Si(OH)4	Chla	WindStress	Rainfall
R4.20190607	R4	2019	6	07/06/2019	1.452	50.799	PAR	Temp	S	NO2	NO3	PO4	Si(OH)4	Chla	WindStress	Rainfall
R1.20190611	R1	2019	6	11/06/2019	1.567	50.799	PAR	Temp	S	NO2	NO3	PO4	Si(OH)4	Chla	WindStress	Rainfall
R4.20190611	R4	2019	6	11/06/2019	1.452	50.799	PAR	Temp	S	NO2	NO3	PO4	Si(OH)4	Chla	WindStress	Rainfall
R1.20190614	R1	2019	6	14/06/2019	1.567	50.799	PAR	Temp	S	NO2	NO3	PO4	Si(OH)4	Chla	WindStress	Rainfall
S1.20190617	S1	2019	6	17/06/2019	1.521	50.688	PAR	Temp	S	NO2	NO3	PO4	Si(OH)4	Chla	WindStress	Rainfall
S2.20190617	S2	2019	6	17/06/2019	1.417	50.688	PAR	Temp	S	NO2	NO3	PO4	Si(OH)4	Chla	WindStress	Rainfall
R1.20190621	R1	2019	6	21/06/2019	1.567	50.799	PAR	Temp	S	NO2	NO3	PO4	Si(OH)4	Chla	WindStress	Rainfall
R2.20190621	R2	2019	6	21/06/2019	1.542	50.799	PAR	Temp	S	NO2	NO3	PO4	Si(OH)4	Chla	WindStress	Rainfall
R1.20190628	R1	2019	6	28/06/2019	1.567	50.799	PAR	Temp	S	NO2	NO3	PO4	Si(OH)4	Chla	WindStress	Rainfall
R1.20190701	R1	2019	7	01/07/2019	1.567	50.799	PAR	Temp	S	NO2	NO3	PO4	Si(OH)4	Chla	WindStress	Rainfall
R4.20190701	R4	2019	7	01/07/2019	1.452	50.799	PAR	Temp	S	NO2	NO3	PO4	Si(OH)4	Chla	WindStress	Rainfall
S1.20190702	S1	2019	7	02/07/2019	1.521	50.688	PAR	Temp	S	NO2	NO3	PO4	Si(OH)4	Chla	WindStress	Rainfall
S2.20190702	S2	2019	7	02/07/2019	1.417	50.688	PAR	Temp	S	NO2	NO3	PO4	Si(OH)4	Chla	WindStress	Rainfall
R1.20190703	R1	2019	7	03/07/2019	1.567	50.799	PAR	Temp	S	NO2	NO3	PO4	Si(OH)4	Chla	WindStress	Rainfall
R2.20190703	R2	2019	7	03/07/2019	1.542	50.799	PAR	Temp	S	NO2	NO3	PO4	Si(OH)4	Chla	WindStress	Rainfall
R4.20190703	R4	2019	7	03/07/2019	1.452	50.799	PAR	Temp	S	NO2	NO3	PO4	Si(OH)4	Chla	WindStress	Rainfall
R1.20190706	R1	2019	7	06/07/2019	1.567	50.799	PAR	Temp	S	NO2	NO3	PO4	Si(OH)4	Chla	WindStress	Rainfall
R4.20190706	R4	2019	7	06/07/2019	1.452	50.799	PAR	Temp	S	NO2	NO3	PO4	Si(OH)4	Chla	WindStress	Rainfall
R1.20190709	R1	2019	7	09/07/2019	1.567	50.799	PAR	Temp	S	NO2	NO3	PO4	Si(OH)4	Chla	WindStress	Rainfall
R2.20190709	R2	2019	7	09/07/2019	1.542	50.799	PAR	Temp	S	NO2	NO3	PO4	Si(OH)4	Chla	WindStress	Rainfall
R1.20190716	R1	2019	7	16/07/2019	1.567	50.799	PAR	Temp	S	NO2	NO3	PO4	Si(OH)4	Chla	WindStress	Rainfall
R2.20190716	R2	2019	7	16/07/2019	1.542	50.799	PAR	Temp	S	NO2	NO3	PO4	Si(OH)4	Chla	WindStress	Rainfall
R4.20190716	R4	2019	7	16/07/2019	1.452	50.799	PAR	Temp	S	NO2	NO3	PO4	Si(OH)4	Chla	WindStress	Rainfall
S1.20190903	S1	2019	9	03/09/2019	1.521	50.688	PAR	Temp	S	NO2	NO3	PO4	Si(OH)4	Chla	WindStress	Rainfall
R1.20190906	R1	2019	9	06/09/2019	1.567	50.799	PAR	Temp	S	NO2	NO3	PO4	Si(OH)4	Chla	WindStress	Rainfall
S1.20190916	S1	2019	9	16/09/2019	1.521	50.688	PAR	Temp	S	NO2	NO3	PO4	Si(OH)4	Chla	WindStress	Rainfall
S2.20190916	S2	2019	9	16/09/2019	1.417	50.688	PAR	Temp	S	NO2	NO3	PO4	Si(OH)4	Chla	WindStress	Rainfall
R1.20190917	R1	2019	9	17/09/2019	1.567	50.799	PAR	Temp	S	NO2	NO3	PO4	Si(OH)4	Chla	WindStress	Rainfall
R2.20190917	R2	2019	9	17/09/2019	1.542	50.799	PAR	Temp	S	NO2	NO3	PO4	Si(OH)4	Chla	WindStress	Rainfall
R4.20190917	R4	2019	9	17/09/2019	1.452	50.799	PAR	Temp	S	NO2	NO3	PO4	Si(OH)4	Chla	WindStress	Rainfall
S1.20190930	S1	2019	9	30/09/2019	1.521	50.688	PAR	Temp	S	NO2	NO3	PO4	Si(OH)4	Chla	WindStress	Rainfall

S2.20190930	S2	2019	9	30/09/2019	1.417	50.688	PAR	Temp	S	NO2	NO3	PO4	Si(OH)4	Chla	WindStress	Rainfall
R1.20191001	R1	2019	10	01/10/2019	1.567	50.799	PAR	Temp	S	NO2	NO3	PO4	Si(OH)4	Chla	WindStress	Rainfall
R1.20191002	R1	2019	10	02/10/2019	1.567	50.799	PAR	Temp	S	NO2	NO3	PO4	Si(OH)4	Chla	WindStress	Rainfall
R1.20191003	R1	2019	10	03/10/2019	1.567	50.799	PAR	Temp	S	NO2	NO3	PO4	Si(OH)4	Chla	WindStress	Rainfall
R2.20191003	R2	2019	10	03/10/2019	1.542	50.799	PAR	Temp	S	NO2	NO3	PO4	Si(OH)4	Chla	WindStress	Rainfall
R4.20191003	R4	2019	10	03/10/2019	1.452	50.799	PAR	Temp	S	NO2	NO3	PO4	Si(OH)4	Chla	WindStress	Rainfall
R1.20191007	R1	2019	10	07/10/2019	1.567	50.799	PAR	Temp	S	NO2	NO3	PO4	Si(OH)4	Chla	WindStress	Rainfall
S1.20191014	S1	2019	10	14/10/2019	1.521	50.688	PAR	Temp	S	NO2	NO3	PO4	Si(OH)4	Chla	WindStress	Rainfall
S2.20191014	S2	2019	10	14/10/2019	1.417	50.688	PAR	Temp	S	NO2	NO3	PO4	Si(OH)4	Chla	WindStress	Rainfall
S1.20191028	S1	2019	10	28/10/2019	1.521	50.688	PAR	Temp	S	NO2	NO3	PO4	Si(OH)4	Chla	WindStress	Rainfall
S2.20191028	S2	2019	10	28/10/2019	1.417	50.688	PAR	Temp	S	NO2	NO3	PO4	Si(OH)4	Chla	WindStress	Rainfall
R1.20191031	R1	2019	10	31/10/2019	1.567	50.799	PAR	Temp	S	NO2	NO3	PO4	Si(OH)4	Chla	WindStress	Rainfall
R2.20191031	R2	2019	10	31/10/2019	1.542	50.799	PAR	Temp	S	NO2	NO3	PO4	Si(OH)4	Chla	WindStress	Rainfall
R4.20191031	R4	2019	10	31/10/2019	1.452	50.799	PAR	Temp	S	NO2	NO3	PO4	Si(OH)4	Chla	WindStress	Rainfall
S1.20191113	S1	2019	11	13/11/2019	1.521	50.688	PAR	Temp	S	NO2	NO3	PO4	Si(OH)4	Chla	WindStress	Rainfall
R1.20191121	R1	2019	11	21/11/2019	1.567	50.799	PAR	Temp	S	NO2	NO3	PO4	Si(OH)4	Chla	WindStress	Rainfall
R2.20191121	R2	2019	11	21/11/2019	1.542	50.799	PAR	Temp	S	NO2	NO3	PO4	Si(OH)4	Chla	WindStress	Rainfall
R4.20191121	R4	2019	11	21/11/2019	1.452	50.799	PAR	Temp	S	NO2	NO3	PO4	Si(OH)4	Chla	WindStress	Rainfall
R1.20191204	R1	2019	12	04/12/2019	1.567	50.799	PAR	Temp	S	NO2	NO3	PO4	Si(OH)4	Chla	WindStress	Rainfall
R2.20191204	R2	2019	12	04/12/2019	1.542	50.799	PAR	Temp	S	NO2	NO3	PO4	Si(OH)4	Chla	WindStress	Rainfall
R4.20191204	R4	2019	12	04/12/2019	1.452	50.799	PAR	Temp	S	NO2	NO3	PO4	Si(OH)4	Chla	WindStress	Rainfall
R1.20191218	R1	2019	12	18/12/2019	1.567	50.799	PAR	Temp	S	NO2	NO3	PO4	Si(OH)4	Chla	WindStress	Rainfall
R1.20200214	R1	2020	2	14/02/2020	1.567	50.799	PAR	Temp	S	NO2	NO3	PO4	Si(OH)4	Chla	WindStress	Rainfall
R2.20200214	R2	2020	2	14/02/2020	1.542	50.799	PAR	Temp	S	NO2	NO3	PO4	Si(OH)4	Chla	WindStress	Rainfall
R4.20200214	R4	2020	2	14/02/2020	1.452	50.799	PAR	Temp	S	NO2	NO3	PO4	Si(OH)4	Chla	WindStress	Rainfall
S1.20200214	S1	2020	2	14/02/2020	1.521	50.688	PAR	Temp	S	NO2	NO3	PO4	Si(OH)4	Chla	WindStress	Rainfall
R1.20200520	R1	2020	5	20/05/2020	1.567	50.799	PAR	Temp	S	NO2	NO3	PO4	Si(OH)4	Chla	WindStress	Rainfall
R2.20200520	R2	2020	5	20/05/2020	1.542	50.799	PAR	Temp	S	NO2	NO3	PO4	Si(OH)4	Chla	WindStress	Rainfall
S1.20200525	S1	2020	5	25/05/2020	1.521	50.688	PAR	Temp	S	NO2	NO3	PO4	Si(OH)4	Chla	WindStress	Rainfall
R1.20200527	R1	2020	5	27/05/2020	1.567	50.799	PAR	Temp	S					Chla	WindStress	Rainfall
R2.20200527	R2	2020	5	27/05/2020	1.542	50.799	PAR	Temp	S					Chla	WindStress	Rainfall
R1.20200528	R1	2020	5	28/05/2020	1.567	50.799	PAR							Chla	WindStress	Rainfall
R1.20200529	R1	2020	5	29/05/2020	1.567	50.799	PAR							Chla	WindStress	Rainfall
S1.20200603	S1	2020	6	03/06/2020	1.521	50.688	PAR	Temp	S	NO2	NO3	PO4	Si(OH)4	Chla	WindStress	Rainfall
S2.20200603	S2	2020	6	03/06/2020	1.417	50.688	PAR	Temp	S	NO2	NO3	PO4	Si(OH)4	Chla	WindStress	Rainfall
R1.20200604	R1	2020	6	04/06/2020	1.567	50.799	PAR	Temp	S	NO2	NO3	PO4	Si(OH)4	Chla	WindStress	Rainfall
R2.20200604	R2	2020	6	04/06/2020	1.542	50.799	PAR	Temp	S	NO2	NO3	PO4	Si(OH)4	Chla	WindStress	Rainfall
R1.20200609	R1	2020	6	09/06/2020	1.567	50.799	PAR			NO2	NO3	PO4	Si(OH)4	Chla	WindStress	Rainfall
R1.20200610	R1	2020	6	10/06/2020	1.567	50.799	PAR	Temp	S	NO2	NO3	PO4	Si(OH)4	Chla	WindStress	Rainfall
R2.20200610	R2	2020	6	10/06/2020	1.542	50.799	PAR	Temp	S	NO2	NO3	PO4	Si(OH)4	Chla	WindStress	Rainfall
R4.20200610	R4	2020	6	10/06/2020	1.452	50.799	PAR	Temp	S	NO2	NO3	PO4	Si(OH)4	Chla	WindStress	Rainfall
R1.20200612	R1	2020	6	12/06/2020	1.567	50.799	PAR	Temp	S	NO2	NO3	PO4	Si(OH)4	Chla	WindStress	Rainfall
R1.20200615	R1	2020	6	15/06/2020	1.567	50.799	PAR	Temp		NO2	NO3	PO4	Si(OH)4	Chla	WindStress	Rainfall
R4.20200615	R4	2020	6	15/06/2020	1.452	50.799	PAR	Temp	S	NO2	NO3	PO4	Si(OH)4	Chla	WindStress	Rainfall
R1.20200616	R1	2020	6	16/06/2020	1.567	50.799	PAR	Temp	S	NO2	NO3	PO4	Si(OH)4	Chla	WindStress	Rainfall
R4.20200616	R4	2020	6	16/06/2020	1.452	50.799	PAR	Temp	S	NO2	NO3	PO4	Si(OH)4	Chla	WindStress	Rainfall
R1.20200617	R1	2020	6	17/06/2020	1.567	50.799	PAR	Temp	S	NO2	NO3	PO4	Si(OH)4	Chla	WindStress	Rainfall
R2.20200617	R2	2020	6	17/06/2020	1.542	50.799	PAR	Temp	S	NO2	NO3	PO4	Si(OH)4	Chla	WindStress	Rainfall

R4.20200617	R4	2020	6	17/06/2020	1.452	50.799	PAR	Temp	S	NO2	NO3	PO4	Si(OH)4	Chla	WindStress	Rainfall
R1.20200618	R1	2020	6	18/06/2020	1.567	50.799	PAR	Temp	S	NO2	NO3	PO4	Si(OH)4	Chla	WindStress	Rainfall
R2.20200618	R2	2020	6	18/06/2020	1.542	50.799	PAR	Temp	S	NO2	NO3	PO4	Si(OH)4	Chla	WindStress	Rainfall
R1.20200619	R1	2020	6	19/06/2020	1.567	50.799	PAR			NO2	NO3	PO4	Si(OH)4	Chla	WindStress	Rainfall
S1.20200623	S1	2020	6	23/06/2020	1.521	50.688	PAR	Temp	S	NO2	NO3	PO4	Si(OH)4	Chla	WindStress	Rainfall
R1.20200624	R1	2020	6	24/06/2020	1.567	50.799	PAR			NO2	NO3	PO4	Si(OH)4	Chla	WindStress	Rainfall
R2.20200624	R2	2020	6	24/06/2020	1.542	50.799	PAR			NO2	NO3	PO4	Si(OH)4	Chla	WindStress	Rainfall
R1.20200625	R1	2020	6	25/06/2020	1.567	50.799	PAR			NO2	NO3	PO4	Si(OH)4	Chla	WindStress	Rainfall
S1.20200707	S1	2020	7	07/07/2020	1.521	50.688	PAR	Temp	S	NO2	NO3	PO4	Si(OH)4	Chla	WindStress	Rainfall
R1.20200715	R1	2020	7	15/07/2020	1.567	50.799	PAR	Temp	S	NO2	NO3	PO4	Si(OH)4	Chla	WindStress	Rainfall
R2.20200715	R2	2020	7	15/07/2020	1.542	50.799	PAR	Temp	S	NO2	NO3	PO4	Si(OH)4	Chla	WindStress	Rainfall
R4.20200715	R4	2020	7	15/07/2020	1.452	50.799	PAR	Temp	S	NO2	NO3	PO4	Si(OH)4	Chla	WindStress	Rainfall
R1.20200721	R1	2020	7	21/07/2020	1.567	50.799	PAR	Temp	S	NO2	NO3	PO4	Si(OH)4	Chla	WindStress	Rainfall
R2.20200721	R2	2020	7	21/07/2020	1.542	50.799	PAR	Temp	S	NO2	NO3	PO4	Si(OH)4	Chla	WindStress	Rainfall
R4.20200721	R4	2020	7	21/07/2020	1.452	50.799	PAR	Temp	S	NO2	NO3	PO4	Si(OH)4	Chla	WindStress	Rainfall
S1.20200722	S1	2020	7	22/07/2020	1.521	50.688	PAR	Temp	S	NO2	NO3	PO4	Si(OH)4	Chla	WindStress	Rainfall
S2.20200722	S2	2020	7	22/07/2020	1.417	50.688	PAR	Temp	S	NO2	NO3	PO4	Si(OH)4	Chla	WindStress	Rainfall
R1.20200730	R1	2020	7	30/07/2020	1.567	50.799	PAR	Temp	S	NO2	NO3	PO4	Si(OH)4	Chla	WindStress	Rainfall
R2.20200730	R2	2020	7	30/07/2020	1.542	50.799	PAR	Temp	S	NO2	NO3	PO4	Si(OH)4	Chla	WindStress	Rainfall
R4.20200730	R4	2020	7	30/07/2020	1.452	50.799	PAR	Temp	S	NO2	NO3	PO4	Si(OH)4	Chla	WindStress	Rainfall
R1.20200901	R1	2020	9	01/09/2020	1.567	50.799	PAR	Temp	S	NO2	NO3	PO4	Si(OH)4	Chla	WindStress	Rainfall
R2.20200901	R2	2020	9	01/09/2020	1.542	50.799	PAR	Temp	S	NO2	NO3	PO4	Si(OH)4	Chla	WindStress	Rainfall
R4.20200901	R4	2020	9	01/09/2020	1.452	50.799	PAR	Temp	S	NO2	NO3	PO4	Si(OH)4	Chla	WindStress	Rainfall
S1.20200902	S1	2020	9	02/09/2020	1.521	50.688	PAR	Temp	S	NO2	NO3	PO4	Si(OH)4	Chla	WindStress	Rainfall
S2.20200902	S2	2020	9	02/09/2020	1.417	50.688	PAR	Temp	S	NO2	NO3	PO4	Si(OH)4	Chla	WindStress	Rainfall
R1.20200904	R1	2020	9	04/09/2020	1.567	50.799	PAR	Temp	S	NO2	NO3	PO4	Si(OH)4	Chla	WindStress	Rainfall
R4.20200904	R4	2020	9	04/09/2020	1.452	50.799	PAR	Temp	S	NO2	NO3	PO4	Si(OH)4	Chla	WindStress	Rainfall
R1.20200907	R1	2020	9	07/09/2020	1.567	50.799	PAR	Temp	S	NO2	NO3	PO4	Si(OH)4	Chla	WindStress	Rainfall
R4.20200907	R4	2020	9	07/09/2020	1.452	50.799	PAR	Temp	S	NO2	NO3	PO4	Si(OH)4	Chla	WindStress	Rainfall
R1.20200911	R1	2020	9	11/09/2020	1.567	50.799	PAR	Temp	S	NO2	NO3	PO4	Si(OH)4	Chla	WindStress	Rainfall
R2.20200911	R2	2020	9	11/09/2020	1.542	50.799	PAR	Temp	S	NO2	NO3	PO4	Si(OH)4	Chla	WindStress	Rainfall
R4.20200911	R4	2020	9	11/09/2020	1.452	50.799	PAR	Temp	S	NO2	NO3	PO4	Si(OH)4	Chla	WindStress	Rainfall
R1.20200914	R1	2020	9	14/09/2020	1.567	50.799	PAR	Temp	S	NO2	NO3	PO4	Si(OH)4	Chla	WindStress	Rainfall
R4.20200914	R4	2020	9	14/09/2020	1.452	50.799	PAR	Temp	S	NO2	NO3	PO4	Si(OH)4	Chla	WindStress	Rainfall
S1.20200916	S1	2020	9	16/09/2020	1.521	50.688	PAR	Temp	S	NO2	NO3	PO4	Si(OH)4	Chla	WindStress	Rainfall
S2.20200916	S2	2020	9	16/09/2020	1.417	50.688	PAR	Temp	S	NO2	NO3	PO4	Si(OH)4	Chla	WindStress	Rainfall
R1.20200917	R1	2020	9	17/09/2020	1.567	50.799	PAR	Temp	S	NO2	NO3	PO4	Si(OH)4	Chla	WindStress	Rainfall
R2.20200917	R2	2020	9	17/09/2020	1.542	50.799	PAR	Temp	S	NO2	NO3	PO4	Si(OH)4	Chla	WindStress	Rainfall
R1.20200929	R1	2020	9	29/09/2020	1.567	50.799	PAR	Temp	S	NO2	NO3	PO4	Si(OH)4	Chla	WindStress	Rainfall
R2.20200929	R2	2020	9	29/09/2020	1.542	50.799	PAR	Temp	S	NO2	NO3	PO4	Si(OH)4	Chla	WindStress	Rainfall
R4.20200929	R4	2020	9	29/09/2020	1.452	50.799	PAR	Temp	S	NO2	NO3	PO4	Si(OH)4	Chla	WindStress	Rainfall
R1.20200930	R1	2020	9	30/09/2020	1.567	50.799	PAR	Temp	S	NO2	NO3	PO4	Si(OH)4	Chla	WindStress	Rainfall
R4.20200930	R4	2020	9	30/09/2020	1.452	50.799	PAR	Temp	S	NO2	NO3	PO4	Si(OH)4	Chla	WindStress	Rainfall
S1.20201001	S1	2020	10	01/10/2020	1.521	50.688	PAR	Temp	S	NO2	NO3	PO4	Si(OH)4	Chla	WindStress	Rainfall
S2.20201001	S2	2020	10	01/10/2020	1.417	50.688	PAR	Temp	S	NO2	NO3	PO4	Si(OH)4	Chla	WindStress	Rainfall
R1.20201002	R1	2020	10	02/10/2020	1.567	50.799	PAR	Temp	S	NO2	NO3	PO4	Si(OH)4	Chla	WindStress	Rainfall
R1.20201009	R1	2020	10	09/10/2020	1.567	50.799	PAR	Temp	S	NO2	NO3	PO4	Si(OH)4	Chla	WindStress	Rainfall
R2.20201009	R2	2020	10	09/10/2020	1.542	50.799	PAR	Temp	S	NO2	NO3	PO4	Si(OH)4	Chla	WindStress	Rainfall

R4.20201009	R4	2020	10	09/10/2020	1.452	50.799	PAR	Temp	S	NO2	NO3	PO4	Si(OH)4	Chla	WindStress	Rainfall
S1.20201015	S1	2020	10	15/10/2020	1.521	50.688	PAR	Temp	S	NO2	NO3	PO4	Si(OH)4	Chla	WindStress	Rainfall
S2.20201015	S2	2020	10	15/10/2020	1.417	50.688	PAR	Temp	S	NO2	NO3	PO4	Si(OH)4	Chla	WindStress	Rainfall
R1.20201016	R1	2020	10	16/10/2020	1.567	50.799	PAR	Temp	S	NO2	NO3	PO4	Si(OH)4	Chla	WindStress	Rainfall
R2.20201016	R2	2020	10	16/10/2020	1.542	50.799	PAR	Temp	S	NO2	NO3	PO4	Si(OH)4	Chla	WindStress	Rainfall
R4.20201016	R4	2020	10	16/10/2020	1.452	50.799	PAR	Temp	S	NO2	NO3	PO4	Si(OH)4	Chla	WindStress	Rainfall
**Number of samples**							**322**	**310**	**##**	**314**	**310**	**315**	**315**	**320**	**322**	**322**

Table S2. List of metabarcoding data (18S rDNA), microscopic counts: diatoms (cell L^{-1}), dinoflagellates (cell L^{-1}), Phaeocystis globosa (cell L^{-1}), cytometric data: pico-nanophytoplankton (cell L^{-1}), cryptophytes (cell L^{-1}) for each date sampled in the eastern Englsih Channel at the SOMLIT and DYPHYRAD stations from March 2016 to October 2020.

	Station	Month	Year	Date	Longitude	Latitude	Metabarcoding	Diatoms_counts	Dinoflagellates_counts	Phaeocystis_counts	PicoNano_counts	Crypto_counts
S1.20160126	S1	1	2016	26/01/2016	1.521	50.688		Diatoms_counts		Phaeocystis_counts	PicoNano_counts	Crypto_counts
S1.20160222	S1	1	2016	22/02/2016	1.521	50.688		Diatoms_counts		Phaeocystis_counts	PicoNano_counts	Crypto_counts
S1.20160307	S1	3	2016	07/03/2016	1.521	50.688	Metabarcoding	Diatoms_counts		Phaeocystis_counts	PicoNano_counts	Crypto_counts
S1.20160322	S1	3	2016	22/03/2016	1.521	50.688	Metabarcoding					
S2.20160322	S2	3	2016	22/03/2016	1.417	50.688	Metabarcoding	Diatoms_counts		Phaeocystis_counts	PicoNano_counts	Crypto_counts
S1.20160405	S1	4	2016	05/04/2016	1.521	50.688	Metabarcoding	Diatoms_counts		Phaeocystis_counts	PicoNano_counts	Crypto_counts
S2.20160405	S2	4	2016	05/04/2016	1.417	50.688	Metabarcoding					
S1.20160420	S1	4	2016	20/04/2016	1.521	50.688	Metabarcoding	Diatoms_counts		Phaeocystis_counts	PicoNano_counts	Crypto_counts
S1.20160509	S1	5	2016	09/05/2016	1.521	50.688	Metabarcoding	Diatoms_counts		Phaeocystis_counts	PicoNano_counts	Crypto_counts
S2.20160509	S2	5	2016	09/05/2016	1.417	50.688	Metabarcoding					
S1.20160523	S1	5	2016	23/05/2016	1.521	50.688	Metabarcoding	Diatoms_counts		Phaeocystis_counts	PicoNano_counts	Crypto_counts
S2.20160523	S2	5	2016	23/05/2016	1.417	50.688	Metabarcoding					
S1.20160606	S1	6	2016	06/06/2016	1.521	50.688	Metabarcoding	Diatoms_counts		Phaeocystis_counts	PicoNano_counts	Crypto_counts
S1.20160621	S1	6	2016	21/06/2016	1.521	50.688	Metabarcoding	Diatoms_counts		Phaeocystis_counts	PicoNano_counts	Crypto_counts
S1.20160706	S1	7	2016	06/07/2016	1.521	50.688	Metabarcoding	Diatoms_counts		Phaeocystis_counts	PicoNano_counts	Crypto_counts
S1.20160721	S1	7	2016	21/07/2016	1.521	50.688	Metabarcoding	Diatoms_counts		Phaeocystis_counts	PicoNano_counts	Crypto_counts
S1.20160902	S1	9	2016	02/09/2016	1.521	50.688	Metabarcoding	Diatoms_counts		Phaeocystis_counts	PicoNano_counts	Crypto_counts
S1.20160919	S1	9	2016	19/09/2016	1.521	50.688	Metabarcoding	Diatoms_counts		Phaeocystis_counts	PicoNano_counts	Crypto_counts
S2.20160919	S2	9	2016	19/09/2016	1.417	50.688	Metabarcoding					
S1.20161003	S1	10	2016	03/10/2016	1.521	50.688	Metabarcoding					
S2.20161003	S2	10	2016	03/10/2016	1.417	50.688	Metabarcoding					
S1.20161103	S1	11	2016	03/11/2016	1.521	50.688	Metabarcoding	Diatoms_counts		Phaeocystis_counts	PicoNano_counts	Crypto_counts
S2.20161103	S2	11	2016	03/11/2016	1.417	50.688	Metabarcoding					
S1.20161114	S1	11	2016	14/11/2016	1.521	50.688	Metabarcoding	Diatoms_counts		Phaeocystis_counts	PicoNano_counts	Crypto_counts
S2.20161114	S2	11	2016	14/11/2016	1.417	50.688	Metabarcoding					
S1.20161201	S1	12	2016	01/12/2016	1.521	50.688	Metabarcoding	Diatoms_counts		Phaeocystis_counts	PicoNano_counts	Crypto_counts

S2.20161201	S2	12	2016	01/12/2016	1.417	50.688	Metabarcoding					
S1.20161212	S1	12	2016	12/12/2016	1.521	50.688	Metabarcoding					
S2.20161212	S2	12	2016	12/12/2016	1.417	50.688	Metabarcoding					
S1.20170130	S1	1	2017	30/01/2017	1.521	50.688	Metabarcoding	Diatoms_counts		Phaeocystis_counts	PicoNano_counts	Crypto_counts
S2.20170130	S2	1	2017	30/01/2017	1.417	50.688	Metabarcoding					
S1.20170214	S1	2	2017	14/02/2017	1.521	50.688	Metabarcoding	Diatoms_counts		Phaeocystis_counts	PicoNano_counts	Crypto_counts
S1.20170313	S1	3	2017	13/03/2017	1.521	50.688	Metabarcoding	Diatoms_counts		Phaeocystis_counts	PicoNano_counts	Crypto_counts
S2.20170313	S2	3	2017	13/03/2017	1.417	50.688	Metabarcoding					
S1.20170327	S1	3	2017	27/03/2017	1.521	50.688	Metabarcoding	Diatoms_counts		Phaeocystis_counts	PicoNano_counts	Crypto_counts
S2.20170327	S2	3	2017	27/03/2017	1.417	50.688	Metabarcoding					
S1.20170411	S1	4	2017	11/04/2017	1.521	50.688	Metabarcoding	Diatoms_counts		Phaeocystis_counts	PicoNano_counts	Crypto_counts
S2.20170411	S2	4	2017	11/04/2017	1.417	50.688	Metabarcoding					
S1.20170510	S1	5	2017	10/05/2017	1.521	50.688	Metabarcoding	Diatoms_counts		Phaeocystis_counts	PicoNano_counts	Crypto_counts
S2.20170510	S2	5	2017	10/05/2017	1.417	50.688	Metabarcoding					
S1.20170524	S1	5	2017	24/05/2017	1.521	50.688	Metabarcoding	Diatoms_counts		Phaeocystis_counts	PicoNano_counts	Crypto_counts
S2.20170524	S2	5	2017	24/05/2017	1.417	50.688	Metabarcoding					
S1.20170627	S1	6	2017	27/06/2017	1.521	50.688	Metabarcoding	Diatoms_counts		Phaeocystis_counts	PicoNano_counts	Crypto_counts
S2.20170627	S2	6	2017	27/06/2017	1.417	50.688	Metabarcoding					
S1.20170711	S1	7	2017	11/07/2017	1.521	50.688	Metabarcoding	Diatoms_counts		Phaeocystis_counts	PicoNano_counts	Crypto_counts
S1.20170724	S1	7	2017	24/07/2017	1.521	50.688	Metabarcoding	Diatoms_counts		Phaeocystis_counts	PicoNano_counts	Crypto_counts
S2.20170724	S2	7	2017	24/07/2017	1.417	50.688	Metabarcoding					
S1.20170907	S1	9	2017	07/09/2017	1.521	50.688	Metabarcoding					
S2.20170907	S2	9	2017	07/09/2017	1.417	50.688	Metabarcoding					
S1.20170918	S1	9	2017	18/09/2017	1.521	50.688	Metabarcoding	Diatoms_counts		Phaeocystis_counts	PicoNano_counts	Crypto_counts
S2.20170918	S2	9	2017	18/09/2017	1.417	50.688	Metabarcoding					
S1.20171004	S1	10	2017	04/10/2017	1.521	50.688	Metabarcoding	Diatoms_counts		Phaeocystis_counts	PicoNano_counts	Crypto_counts
S1.20171018	S1	10	2017	18/10/2017	1.521	50.688	Metabarcoding	Diatoms_counts		Phaeocystis_counts	PicoNano_counts	Crypto_counts
S2.20171018	S2	10	2017	18/10/2017	1.417	50.688	Metabarcoding					
S1.20171102	S1	11	2017	02/11/2017	1.521	50.688	Metabarcoding	Diatoms_counts		Phaeocystis_counts	PicoNano_counts	Crypto_counts
S1.20171116	S1	11	2017	16/11/2017	1.521	50.688	Metabarcoding	Diatoms_counts		Phaeocystis_counts	PicoNano_counts	Crypto_counts
S2.20171116	S2	11	2017	16/11/2017	1.417	50.688	Metabarcoding					

S1.20171204	S1	12	2017	04/12/2017	1.521	50.688		Diatoms_counts		Phaeocystis_counts	PicoNano_counts	Crypto_counts
S1.20171219	S1	12	2017	19/12/2017	1.521	50.688	Metabarcoding	Diatoms_counts		Phaeocystis_counts		
S2.20171219	S2	12	2017	19/12/2017	1.417	50.688	Metabarcoding					
R1.20180207	R1	2	2018	07/02/2018	1.567	50.799	Metabarcoding	Diatoms_counts		Phaeocystis_counts	PicoNano_counts	Crypto_counts
R2.20180207	R2	2	2018	07/02/2018	1.542	50.799	Metabarcoding	Diatoms_counts		Phaeocystis_counts	PicoNano_counts	Crypto_counts
R4.20180207	R4	2	2018	07/02/2018	1.452	50.799	Metabarcoding	Diatoms_counts		Phaeocystis_counts	PicoNano_counts	Crypto_counts
S1.20180216	S1	2	2018	16/02/2018	1.521	50.688	Metabarcoding	Diatoms_counts		Phaeocystis_counts	PicoNano_counts	Crypto_counts
S2.20180216	S2	2	2018	16/02/2018	1.417	50.688	Metabarcoding	Diatoms_counts		Phaeocystis_counts	PicoNano_counts	Crypto_counts
R1.20180228	R1	2	2018	28/02/2018	1.567	50.799	Metabarcoding	Diatoms_counts		Phaeocystis_counts	PicoNano_counts	Crypto_counts
R2.20180228	R2	2	2018	28/02/2018	1.542	50.799	Metabarcoding	Diatoms_counts		Phaeocystis_counts	PicoNano_counts	Crypto_counts
R4.20180228	R4	2	2018	28/02/2018	1.452	50.799	Metabarcoding	Diatoms_counts		Phaeocystis_counts	PicoNano_counts	Crypto_counts
S1.20180302	S1	3	2018	02/03/2018	1.521	50.688		Diatoms_counts		Phaeocystis_counts	PicoNano_counts	Crypto_counts
S2.20180302	S2	3	2018	02/03/2018	1.417	50.688		Diatoms_counts		Phaeocystis_counts	PicoNano_counts	Crypto_counts
R1.20180315	R1	3	2018	15/03/2018	1.567	50.799	Metabarcoding	Diatoms_counts		Phaeocystis_counts	PicoNano_counts	Crypto_counts
R2.20180315	R2	3	2018	15/03/2018	1.542	50.799	Metabarcoding	Diatoms_counts		Phaeocystis_counts	PicoNano_counts	Crypto_counts
R4.20180315	R4	3	2018	15/03/2018	1.452	50.799	Metabarcoding	Diatoms_counts		Phaeocystis_counts	PicoNano_counts	Crypto_counts
S1.20180319	S1	3	2018	19/03/2018	1.521	50.688	Metabarcoding	Diatoms_counts	Dinoflagellates_counts	Phaeocystis_counts	PicoNano_counts	Crypto_counts
S2.20180319	S2	3	2018	19/03/2018	1.417	50.688	Metabarcoding					
R1.20180322	R1	3	2018	22/03/2018	1.567	50.799	Metabarcoding	Diatoms_counts		Phaeocystis_counts	PicoNano_counts	Crypto_counts
R1.20180329	R1	3	2018	29/03/2018	1.567	50.799	Metabarcoding	Diatoms_counts		Phaeocystis_counts	PicoNano_counts	Crypto_counts
R2.20180329	R2	3	2018	29/03/2018	1.542	50.799	Metabarcoding	Diatoms_counts		Phaeocystis_counts	PicoNano_counts	Crypto_counts
R1.20180411	R1	4	2018	11/04/2018	1.567	50.799	Metabarcoding	Diatoms_counts	Dinoflagellates_counts	Phaeocystis_counts	PicoNano_counts	Crypto_counts
R2.20180411	R2	4	2018	11/04/2018	1.542	50.799	Metabarcoding	Diatoms_counts	Dinoflagellates_counts	Phaeocystis_counts	PicoNano_counts	Crypto_counts
R4.20180411	R4	4	2018	11/04/2018	1.452	50.799	Metabarcoding	Diatoms_counts	Dinoflagellates_counts	Phaeocystis_counts	PicoNano_counts	Crypto_counts
S1.20180416	S1	4	2018	16/04/2018	1.521	50.688		Diatoms_counts	Dinoflagellates_counts	Phaeocystis_counts	PicoNano_counts	Crypto_counts
S1.20180416	S2	4	2018	16/04/2018	1.417	50.688		Diatoms_counts	Dinoflagellates_counts	Phaeocystis_counts	PicoNano_counts	Crypto_counts
R1.20180417	R1	4	2018	17/04/2018	1.567	50.799	Metabarcoding	Diatoms_counts	Dinoflagellates_counts	Phaeocystis_counts	PicoNano_counts	Crypto_counts
R2.20180417	R2	4	2018	17/04/2018	1.542	50.799	Metabarcoding	Diatoms_counts	Dinoflagellates_counts	Phaeocystis_counts	PicoNano_counts	Crypto_counts
R4.20180417	R4	4	2018	17/04/2018	1.452	50.799	Metabarcoding					
S1.20180503	S1	5	2018	03/05/2018	1.521	50.688		Diatoms_counts	Dinoflagellates_counts	Phaeocystis_counts	PicoNano_counts	Crypto_counts
R1.20180504	R1	5	2018	04/05/2018	1.567	50.799		Diatoms_counts	Dinoflagellates_counts	Phaeocystis_counts	PicoNano_counts	Crypto_counts

R2.20180504	R2	5	2018	04/05/2018	1.542	50.799	Metabarcoding	Diatoms_counts	Dinoflagellates_counts	Phaeocystis_counts	PicoNano_counts	Crypto_counts
R4.20180504	R4	5	2018	04/05/2018	1.452	50.799		Diatoms_counts		Phaeocystis_counts	PicoNano_counts	Crypto_counts
R1.20180511	R1	5	2018	11/05/2018	1.567	50.799		Diatoms_counts	Dinoflagellates_counts	Phaeocystis_counts	PicoNano_counts	Crypto_counts
R1.20180516	R1	5	2018	16/05/2018	1.567	50.799	Metabarcoding	Diatoms_counts	Dinoflagellates_counts	Phaeocystis_counts	PicoNano_counts	Crypto_counts
S1.20180523	S1	5	2018	23/05/2018	1.521	50.688	Metabarcoding					
S2.20180523	S2	5	2018	24/05/2018	1.417	50.688	Metabarcoding					
S1.20180529	S1	5	2018	29/05/2018	1.521	50.688	Metabarcoding	Diatoms_counts	Dinoflagellates_counts	Phaeocystis_counts	PicoNano_counts	Crypto_counts
S2.20180529	S2	5	2018	29/05/2018	1.417	50.688	Metabarcoding	Diatoms_counts	Dinoflagellates_counts	Phaeocystis_counts	PicoNano_counts	Crypto_counts
R1.20180531	R1	5	2018	31/05/2018	1.567	50.799	Metabarcoding	Diatoms_counts	Dinoflagellates_counts	Phaeocystis_counts	PicoNano_counts	Crypto_counts
R2.20180531	R2	5	2018	31/05/2018	1.542	50.799	Metabarcoding	Diatoms_counts	Dinoflagellates_counts	Phaeocystis_counts	PicoNano_counts	Crypto_counts
R4.20180531	R4	5	2018	31/05/2018	1.452	50.799	Metabarcoding	Diatoms_counts	Dinoflagellates_counts	Phaeocystis_counts	PicoNano_counts	Crypto_counts
R1.20180605	R1	6	2018	05/06/2018	1.567	50.799	Metabarcoding	Diatoms_counts	Dinoflagellates_counts	Phaeocystis_counts	PicoNano_counts	Crypto_counts
R1.20180606	R1	6	2018	06/06/2018	1.567	50.799	Metabarcoding					
R1.20180607	R1	6	2018	07/06/2018	1.567	50.799	Metabarcoding	Diatoms_counts	Dinoflagellates_counts	Phaeocystis_counts	PicoNano_counts	Crypto_counts
R2.20180607	R2	6	2018	07/06/2018	1.542	50.799	Metabarcoding	Diatoms_counts	Dinoflagellates_counts	Phaeocystis_counts	PicoNano_counts	Crypto_counts
R4.20180607	R4	6	2018	07/06/2018	1.452	50.799	Metabarcoding	Diatoms_counts	Dinoflagellates_counts	Phaeocystis_counts	PicoNano_counts	Crypto_counts
R1.20180608	R1	6	2018	08/06/2018	1.567	50.799	Metabarcoding					
S1.20180612	S1	6	2018	12/06/2018	1.521	50.688	Metabarcoding	Diatoms_counts	Dinoflagellates_counts	Phaeocystis_counts	PicoNano_counts	Crypto_counts
R2.20180613	R2	6	2018	13/06/2018	1.542	50.799	Metabarcoding	Diatoms_counts	Dinoflagellates_counts	Phaeocystis_counts	PicoNano_counts	Crypto_counts
R1.20180613	R1	6	2018	13/06/2018	1.567	50.799	Metabarcoding	Diatoms_counts	Dinoflagellates_counts	Phaeocystis_counts	PicoNano_counts	Crypto_counts
R4.20180613	R4	6	2018	13/06/2018	1.452	50.799	Metabarcoding	Diatoms_counts	Dinoflagellates_counts	Phaeocystis_counts	PicoNano_counts	Crypto_counts
R1.20180618	R1	6	2018	18/06/2018	1.567	50.799	Metabarcoding	Diatoms_counts	Dinoflagellates_counts	Phaeocystis_counts	PicoNano_counts	Crypto_counts
R4.20180620	R4	6	2018	20/06/2018	1.452	50.799	Metabarcoding	Diatoms_counts	Dinoflagellates_counts	Phaeocystis_counts		
R1.20180620	R1	6	2018	20/06/2018	1.567	50.799	Metabarcoding	Diatoms_counts	Dinoflagellates_counts	Phaeocystis_counts	PicoNano_counts	Crypto_counts
R1.20180622	R1	6	2018	22/06/2018	1.567	50.799	Metabarcoding	Diatoms_counts	Dinoflagellates_counts	Phaeocystis_counts	PicoNano_counts	Crypto_counts
R2.20180622	R2	6	2018	22/06/2018	1.542	50.799		Diatoms_counts	Dinoflagellates_counts	Phaeocystis_counts	PicoNano_counts	Crypto_counts
R4.20180622	R4	6	2018	22/06/2018	1.452	50.799	Metabarcoding	Diatoms_counts	Dinoflagellates_counts	Phaeocystis_counts	PicoNano_counts	Crypto_counts
R1.20180625	R1	6	2018	25/06/2018	1.567	50.799	Metabarcoding	Diatoms_counts	Dinoflagellates_counts	Phaeocystis_counts	PicoNano_counts	Crypto_counts
R1.20180626	R1	6	2018	26/06/2018	1.567	50.799	Metabarcoding	Diatoms_counts	Dinoflagellates_counts	Phaeocystis_counts	PicoNano_counts	Crypto_counts
R1.20180627	R1	6	2018	27/06/2018	1.567	50.799	Metabarcoding	Diatoms_counts	Dinoflagellates_counts	Phaeocystis_counts	PicoNano_counts	Crypto_counts
R2.20180627	R2	6	2018	27/06/2018	1.542	50.799	Metabarcoding	Diatoms_counts	Dinoflagellates_counts	Phaeocystis_counts	PicoNano_counts	Crypto_counts

R4.20180627	R4	6	2018	27/06/2018	1.452	50.799	Metabarcoding	Diatoms_counts	Dinoflagellates_counts	Phaeocystis_counts	PicoNano_counts	Crypto_counts
R1.20180628	R1	6	2018	28/06/2018	1.567	50.799	Metabarcoding	Diatoms_counts		Phaeocystis_counts	PicoNano_counts	Crypto_counts
R4.20180628	R4	6	2018	28/06/2018	1.452	50.799	Metabarcoding	Diatoms_counts		Phaeocystis_counts	PicoNano_counts	Crypto_counts
S1.20180628	S1	6	2018	28/06/2018	1.521	50.688		Diatoms_counts		Phaeocystis_counts	PicoNano_counts	Crypto_counts
R1.20180629	R1	6	2018	29/06/2018	1.567	50.799	Metabarcoding	Diatoms_counts	Dinoflagellates_counts	Phaeocystis_counts	PicoNano_counts	Crypto_counts
R1.20180706	R1	7	2018	06/07/2018	1.567	50.799	Metabarcoding	Diatoms_counts	Dinoflagellates_counts	Phaeocystis_counts	PicoNano_counts	Crypto_counts
R4.20180706	R4	7	2018	07/07/2018	1.452	50.799		Diatoms_counts	Dinoflagellates_counts	Phaeocystis_counts	PicoNano_counts	Crypto_counts
R1.20180711	R1	7	2018	11/07/2018	1.567	50.799	Metabarcoding	Diatoms_counts	Dinoflagellates_counts	Phaeocystis_counts	PicoNano_counts	Crypto_counts
S1.20180712	S1	7	2018	12/07/2018	1.521	50.688	Metabarcoding	Diatoms_counts		Phaeocystis_counts	PicoNano_counts	Crypto_counts
S2.20180712	S2	7	2018	12/07/2018	1.417	50.688	Metabarcoding	Diatoms_counts		Phaeocystis_counts	PicoNano_counts	Crypto_counts
S1.20180727	S1	7	2018	27/07/2018	1.521	50.688	Metabarcoding					
S2.20180727	S2	7	2018	27/07/2018	1.417	50.688	Metabarcoding	Diatoms_counts	Dinoflagellates_counts	Phaeocystis_counts	PicoNano_counts	Crypto_counts
R1.20180828	R1	8	2018	28/08/2018	1.567	50.799	Metabarcoding					
R2.20180828	R2	8	2018	28/08/2018	1.542	50.799	Metabarcoding	Diatoms_counts	Dinoflagellates_counts	Phaeocystis_counts	PicoNano_counts	Crypto_counts
R4.20180828	R4	8	2018	28/08/2018	1.452	50.799	Metabarcoding	Diatoms_counts		Phaeocystis_counts	PicoNano_counts	Crypto_counts
R2.20180925	R2	9	2018	25/09/2018	1.542	50.799	Metabarcoding	Diatoms_counts	Dinoflagellates_counts	Phaeocystis_counts	PicoNano_counts	Crypto_counts
R1.20180925	R1	9	2018	25/09/2018	1.567	50.799	Metabarcoding	Diatoms_counts	Dinoflagellates_counts	Phaeocystis_counts	PicoNano_counts	Crypto_counts
R2.20180925	R2	9	2018	25/09/2018	1.542	50.799		Diatoms_counts	Dinoflagellates_counts	Phaeocystis_counts	PicoNano_counts	Crypto_counts
R4.20180925	R4	9	2018	25/09/2018	1.452	50.799	Metabarcoding					
S1.20180927	S1	9	2018	27/09/2018	1.521	50.688		Diatoms_counts	Dinoflagellates_counts	Phaeocystis_counts	PicoNano_counts	Crypto_counts
S2.20180927	S2	9	2018	27/09/2018	1.417	50.688		Diatoms_counts	Dinoflagellates_counts	Phaeocystis_counts	PicoNano_counts	Crypto_counts
S1.20181008	S1	10	2018	08/10/2018	1.521	50.688		Diatoms_counts	Dinoflagellates_counts	Phaeocystis_counts		
S2.20181008	S2	10	2018	08/10/2018	1.417	50.688		Diatoms_counts	Dinoflagellates_counts	Phaeocystis_counts		
R1.20181011	R1	10	2018	11/10/2018	1.567	50.799	Metabarcoding	Diatoms_counts	Dinoflagellates_counts	Phaeocystis_counts	PicoNano_counts	Crypto_counts
R1.20181019	R1	10	2018	19/10/2018	1.567	50.799		Diatoms_counts		Phaeocystis_counts	PicoNano_counts	Crypto_counts
R2.20181019	R2	10	2018	19/10/2018	1.542	50.799	Metabarcoding	Diatoms_counts		Phaeocystis_counts	PicoNano_counts	Crypto_counts
R4.20181019	R4	10	2018	19/10/2018	1.452	50.799	Metabarcoding	Diatoms_counts		Phaeocystis_counts	PicoNano_counts	Crypto_counts
S1.20181024	S1	10	2018	24/10/2018	1.521	50.688		Diatoms_counts	Dinoflagellates_counts	Phaeocystis_counts	PicoNano_counts	Crypto_counts
S2.20181024	S2	10	2018	24/10/2018	1.417	50.688		Diatoms_counts	Dinoflagellates_counts	Phaeocystis_counts	PicoNano_counts	Crypto_counts
R1.20181025	R1	10	2018	25/10/2018	1.567	50.799	Metabarcoding	Diatoms_counts	Dinoflagellates_counts	Phaeocystis_counts	PicoNano_counts	Crypto_counts
R2.20181025	R2	10	2018	25/10/2018	1.542	50.799		Diatoms_counts	Dinoflagellates_counts	Phaeocystis_counts	PicoNano_counts	Crypto_counts

R4.20181025	R4	10	2018	25/10/2018	1.452	50.799		Diatoms_counts	Dinoflagellates_counts	Phaeocystis_counts	PicoNano_counts	Crypto_counts
R1.20181114	R1	11	2018	14/11/2018	1.567	50.799	Metabarcoding	Diatoms_counts	Dinoflagellates_counts	Phaeocystis_counts	PicoNano_counts	Crypto_counts
R1.20181121	R1	11	2018	21/11/2018	1.567	50.799	Metabarcoding	Diatoms_counts	Dinoflagellates_counts	Phaeocystis_counts	PicoNano_counts	Crypto_counts
R2.20181121	R2	11	2018	21/11/2018	1.542	50.799	Metabarcoding	Diatoms_counts	Dinoflagellates_counts	Phaeocystis_counts	PicoNano_counts	Crypto_counts
S1.20181122	S1	11	2018	22/11/2018	1.521	50.688	Metabarcoding	Diatoms_counts	Dinoflagellates_counts	Phaeocystis_counts	PicoNano_counts	Crypto_counts
S2.20181122	S2	11	2018	22/11/2018	1.417	50.688	Metabarcoding	Diatoms_counts		Phaeocystis_counts	PicoNano_counts	Crypto_counts
S1.20181205	S1	12	2018	05/12/2018	1.521	50.688	Metabarcoding	Diatoms_counts	Dinoflagellates_counts	Phaeocystis_counts	PicoNano_counts	Crypto_counts
R1.20181206	R1	12	2018	06/12/2018	1.567	50.799	Metabarcoding	Diatoms_counts	Dinoflagellates_counts	Phaeocystis_counts	PicoNano_counts	Crypto_counts
R2.20181206	R2	12	2018	06/12/2018	1.542	50.799	Metabarcoding	Diatoms_counts	Dinoflagellates_counts	Phaeocystis_counts	PicoNano_counts	Crypto_counts
R1.20181213	R1	12	2018	13/12/2018	1.567	50.799	Metabarcoding	Diatoms_counts	Dinoflagellates_counts	Phaeocystis_counts	PicoNano_counts	Crypto_counts
R2.20181213	R2	12	2018	13/12/2018	1.542	50.799	Metabarcoding	Diatoms_counts	Dinoflagellates_counts	Phaeocystis_counts	PicoNano_counts	Crypto_counts
R4.20181213	R4	12	2018	13/12/2018	1.452	50.799	Metabarcoding	Diatoms_counts		Phaeocystis_counts	PicoNano_counts	Crypto_counts
R1.20190118	R1	1	2019	18/01/2019	1.567	50.799	Metabarcoding	Diatoms_counts	Dinoflagellates_counts	Phaeocystis_counts	PicoNano_counts	Crypto_counts
R2.20190118	R2	1	2019	18/01/2019	1.542	50.799	Metabarcoding	Diatoms_counts	Dinoflagellates_counts	Phaeocystis_counts	PicoNano_counts	Crypto_counts
R1.20190124	R1	1	2019	24/01/2019	1.567	50.799	Metabarcoding	Diatoms_counts	Dinoflagellates_counts	Phaeocystis_counts		
R2.20190124	R2	1	2019	24/01/2019	1.542	50.799	Metabarcoding	Diatoms_counts	Dinoflagellates_counts	Phaeocystis_counts	PicoNano_counts	Crypto_counts
R4.20190124	R4	1	2019	24/01/2019	1.452	50.799	Metabarcoding					
R2.20190214	R2	2	2019	14/02/2019	1.542	50.799	Metabarcoding					
S1.20190219	S1	2	2019	19/02/2019	1.521	50.688	Metabarcoding	Diatoms_counts	Dinoflagellates_counts	Phaeocystis_counts	PicoNano_counts	Crypto_counts
S2.20190219	S2	2	2019	19/02/2019	1.417	50.688	Metabarcoding	Diatoms_counts	Dinoflagellates_counts	Phaeocystis_counts	PicoNano_counts	Crypto_counts
R1.20190222	R1	2	2019	22/02/2019	1.567	50.799	Metabarcoding	Diatoms_counts	Dinoflagellates_counts	Phaeocystis_counts	PicoNano_counts	Crypto_counts
R2.20190222	R2	2	2019	22/02/2019	1.542	50.799	Metabarcoding	Diatoms_counts	Dinoflagellates_counts	Phaeocystis_counts	PicoNano_counts	Crypto_counts
R4.20190222	R4	2	2019	22/02/2019	1.452	50.799	Metabarcoding	Diatoms_counts	Dinoflagellates_counts	Phaeocystis_counts	PicoNano_counts	Crypto_counts
S1.20190319	S1	3	2019	19/03/2019	1.521	50.688		Diatoms_counts	Dinoflagellates_counts	Phaeocystis_counts	PicoNano_counts	Crypto_counts
S2.20190319	S2	3	2019	19/03/2019	1.417	50.688		Diatoms_counts		Phaeocystis_counts	PicoNano_counts	Crypto_counts
R1.20190322	R1	3	2019	22/03/2019	1.567	50.799	Metabarcoding	Diatoms_counts	Dinoflagellates_counts	Phaeocystis_counts		
R2.20190322	R2	3	2019	22/03/2019	1.542	50.799	Metabarcoding	Diatoms_counts	Dinoflagellates_counts	Phaeocystis_counts		
R4.20190322	R4	3	2019	22/03/2019	1.452	50.799	Metabarcoding	Diatoms_counts	Dinoflagellates_counts	Phaeocystis_counts		
R1.20190327	R1	3	2019	27/03/2019	1.567	50.799	Metabarcoding	Diatoms_counts	Dinoflagellates_counts	Phaeocystis_counts	PicoNano_counts	Crypto_counts
R2.20190327	R2	3	2019	27/03/2019	1.542	50.799	Metabarcoding	Diatoms_counts	Dinoflagellates_counts	Phaeocystis_counts	PicoNano_counts	Crypto_counts
R4.20190327	R4	3	2019	27/03/2019	1.452	50.799	Metabarcoding	Diatoms_counts	Dinoflagellates_counts	Phaeocystis_counts	PicoNano_counts	Crypto_counts

S1.20190404	S1	4	2019	04/04/2019	1.521	50.688	Metabarcoding	Diatoms_counts	Dinoflagellates_counts	Phaeocystis_counts	PicoNano_counts	Crypto_counts
R1.20190405	R1	4	2019	05/04/2019	1.567	50.799	Metabarcoding	Diatoms_counts	Dinoflagellates_counts	Phaeocystis_counts	PicoNano_counts	Crypto_counts
R1.20190411	R1	4	2019	11/04/2019	1.567	50.799	Metabarcoding	Diatoms_counts		Phaeocystis_counts	PicoNano_counts	Crypto_counts
R2.20190411	R2	4	2019	11/04/2019	1.542	50.799	Metabarcoding	Diatoms_counts	Dinoflagellates_counts	Phaeocystis_counts	PicoNano_counts	Crypto_counts
R1.20190416	R1	4	2019	16/04/2019	1.567	50.799	Metabarcoding	Diatoms_counts	Dinoflagellates_counts	Phaeocystis_counts	PicoNano_counts	Crypto_counts
R2.20190416	R2	4	2019	16/04/2019	1.542	50.799	Metabarcoding	Diatoms_counts		Phaeocystis_counts	PicoNano_counts	Crypto_counts
R4.20190416	R4	4	2019	16/04/2019	1.452	50.799	Metabarcoding					
S1.20190417	S1	4	2019	17/04/2019	1.521	50.688	Metabarcoding	Diatoms_counts	Dinoflagellates_counts	Phaeocystis_counts	PicoNano_counts	Crypto_counts
S2.20190417	S2	4	2019	17/04/2019	1.417	50.688	Metabarcoding	Diatoms_counts		Phaeocystis_counts	PicoNano_counts	Crypto_counts
R1.20190426	R1	4	2019	26/04/2019	1.567	50.799	Metabarcoding	Diatoms_counts		Phaeocystis_counts	PicoNano_counts	Crypto_counts
S1.20190516	S1	5	2019	16/05/2019	1.521	50.688	Metabarcoding	Diatoms_counts	Dinoflagellates_counts	Phaeocystis_counts	PicoNano_counts	Crypto_counts
S2.20190516	S2	5	2019	16/05/2019	1.417	50.688	Metabarcoding	Diatoms_counts	Dinoflagellates_counts	Phaeocystis_counts	PicoNano_counts	Crypto_counts
S1.20190603	S1	6	2019	03/06/2019	1.521	50.688	Metabarcoding	Diatoms_counts	Dinoflagellates_counts	Phaeocystis_counts	PicoNano_counts	Crypto_counts
R1.20190605	R1	6	2019	05/06/2019	1.567	50.799		Diatoms_counts	Dinoflagellates_counts	Phaeocystis_counts	PicoNano_counts	Crypto_counts
R1.20190607	R1	6	2019	07/06/2019	1.567	50.799		Diatoms_counts	Dinoflagellates_counts	Phaeocystis_counts	PicoNano_counts	Crypto_counts
R4.20190607	R4	6	2019	07/06/2019	1.452	50.799	Metabarcoding	Diatoms_counts	Dinoflagellates_counts	Phaeocystis_counts	PicoNano_counts	Crypto_counts
R1.20190611	R1	6	2019	11/06/2019	1.567	50.799	Metabarcoding	Diatoms_counts	Dinoflagellates_counts	Phaeocystis_counts	PicoNano_counts	Crypto_counts
R4.20190611	R4	6	2019	11/06/2019	1.452	50.799	Metabarcoding	Diatoms_counts	Dinoflagellates_counts	Phaeocystis_counts	PicoNano_counts	Crypto_counts
R1.20190614	R1	6	2019	14/06/2019	1.567	50.799	Metabarcoding	Diatoms_counts	Dinoflagellates_counts	Phaeocystis_counts		
S1.20190617	S1	6	2019	17/06/2019	1.521	50.688	Metabarcoding	Diatoms_counts	Dinoflagellates_counts	Phaeocystis_counts	PicoNano_counts	
S2.20190617	S2	6	2019	17/06/2019	1.417	50.688		Diatoms_counts		Phaeocystis_counts	PicoNano_counts	Crypto_counts
R1.20190621	R1	6	2019	21/06/2019	1.567	50.799	Metabarcoding	Diatoms_counts	Dinoflagellates_counts	Phaeocystis_counts	PicoNano_counts	Crypto_counts
R2.20190621	R2	6	2019	21/06/2019	1.542	50.799		Diatoms_counts	Dinoflagellates_counts	Phaeocystis_counts	PicoNano_counts	Crypto_counts
S1.20190628	S1	6	2019	28/06/2019	1.521	50.688	Metabarcoding	Diatoms_counts	Dinoflagellates_counts	Phaeocystis_counts		
R1.20190701	R1	7	2019	01/07/2019	1.567	50.799		Diatoms_counts	Dinoflagellates_counts	Phaeocystis_counts	PicoNano_counts	Crypto_counts
R4.20190701	R4	7	2019	01/07/2019	1.452	50.799	Metabarcoding	Diatoms_counts	Dinoflagellates_counts	Phaeocystis_counts	PicoNano_counts	Crypto_counts
S1.20190702	S1	7	2019	02/07/2019	1.521	50.688	Metabarcoding	Diatoms_counts	Dinoflagellates_counts	Phaeocystis_counts	PicoNano_counts	Crypto_counts
S2.20190702	S2	7	2019	02/07/2019	1.417	50.688	Metabarcoding	Diatoms_counts	Dinoflagellates_counts	Phaeocystis_counts	PicoNano_counts	Crypto_counts
R1.20190703	R1	7	2019	03/07/2019	1.567	50.799	Metabarcoding	Diatoms_counts	Dinoflagellates_counts	Phaeocystis_counts	PicoNano_counts	Crypto_counts
R2.20190703	R2	7	2019	03/07/2019	1.542	50.799		Diatoms_counts		Phaeocystis_counts		
R4.20190703	R4	7	2019	03/07/2019	1.452	50.799	Metabarcoding	Diatoms_counts	Dinoflagellates_counts	Phaeocystis_counts	PicoNano_counts	Crypto_counts

R1.20190706	R1	7	2019	06/07/2019	1.567	50.799	Metabarcoding	Diatoms_counts	Dinoflagellates_counts	Phaeocystis_counts	PicoNano_counts	Crypto_counts
R4.20190706	R4	7	2019	06/07/2019	1.452	50.799		Diatoms_counts	Dinoflagellates_counts	Phaeocystis_counts	PicoNano_counts	Crypto_counts
R1.20190709	R1	7	2019	09/07/2019	1.567	50.799	Metabarcoding	Diatoms_counts		Phaeocystis_counts	PicoNano_counts	Crypto_counts
R4.20190709	R4	7	2019	09/07/2019	1.452	50.799	Metabarcoding					
R1.20190716	R1	7	2019	16/07/2019	1.567	50.799	Metabarcoding	Diatoms_counts	Dinoflagellates_counts	Phaeocystis_counts		
R2.20190716	R2	7	2019	16/07/2019	1.542	50.799	Metabarcoding	Diatoms_counts	Dinoflagellates_counts	Phaeocystis_counts	PicoNano_counts	Crypto_counts
R4.20190716	R4	7	2019	16/07/2019	1.452	50.799	Metabarcoding	Diatoms_counts	Dinoflagellates_counts	Phaeocystis_counts	PicoNano_counts	Crypto_counts
S1.20190903	S1	9	2019	03/09/2019	1.521	50.688	Metabarcoding	Diatoms_counts		Phaeocystis_counts	PicoNano_counts	Crypto_counts
R1.20190906	R1	9	2019	06/09/2019	1.567	50.799	Metabarcoding	Diatoms_counts	Dinoflagellates_counts	Phaeocystis_counts	PicoNano_counts	Crypto_counts
S1.20190916	S1	9	2019	16/09/2019	1.521	50.688	Metabarcoding					
S2.20190916	S2	9	2019	16/09/2019	1.417	50.688	Metabarcoding					
R1.20190917	R1	9	2019	17/09/2019	1.567	50.799	Metabarcoding	Diatoms_counts	Dinoflagellates_counts	Phaeocystis_counts	PicoNano_counts	Crypto_counts
R2.20190917	R2	9	2019	17/09/2019	1.542	50.799	Metabarcoding	Diatoms_counts		Phaeocystis_counts	PicoNano_counts	Crypto_counts
R4.20190917	R4	9	2019	17/09/2019	1.452	50.799	Metabarcoding	Diatoms_counts	Dinoflagellates_counts	Phaeocystis_counts	PicoNano_counts	Crypto_counts
S1.20190930	S1	9	2019	30/09/2019	1.521	50.688	Metabarcoding	Diatoms_counts		Phaeocystis_counts	PicoNano_counts	Crypto_counts
S2.20190930	S2		2019	30/09/2019	1.417	50.688		Diatoms_counts	Dinoflagellates_counts	Phaeocystis_counts	PicoNano_counts	Crypto_counts
R1.20191001	R1	10	2019	01/10/2019	1.567	50.799	Metabarcoding					
R1.20191002	R1	10	2019	02/10/2019	1.567	50.799	Metabarcoding	Diatoms_counts	Dinoflagellates_counts	Phaeocystis_counts	PicoNano_counts	Crypto_counts
R1.20191003	R1	10	2019	03/10/2019	1.567	50.799	Metabarcoding	Diatoms_counts	Dinoflagellates_counts	Phaeocystis_counts	PicoNano_counts	Crypto_counts
R2.20191003	R2	10	2019	03/10/2019	1.542	50.799	Metabarcoding	Diatoms_counts	Dinoflagellates_counts	Phaeocystis_counts	PicoNano_counts	Crypto_counts
R4.20191003	R4	10	2019	03/10/2019	1.452	50.799	Metabarcoding	Diatoms_counts	Dinoflagellates_counts	Phaeocystis_counts	PicoNano_counts	Crypto_counts
R1.20191007	R1	10	2019	07/10/2019	1.567	50.799	Metabarcoding	Diatoms_counts	Dinoflagellates_counts	Phaeocystis_counts	PicoNano_counts	Crypto_counts
S1.20191014	S1	10	2019	14/10/2019	1.521	50.688	Metabarcoding	Diatoms_counts		Phaeocystis_counts	PicoNano_counts	Crypto_counts
S2.20191014	S2	10	2019	14/10/2019	1.417	50.688	Metabarcoding	Diatoms_counts		Phaeocystis_counts	PicoNano_counts	Crypto_counts
S1.20191028	S1	10	2019	28/10/2019	1.521	50.688	Metabarcoding	Diatoms_counts		Phaeocystis_counts	PicoNano_counts	Crypto_counts
S2.20191028	S2	10	2019	28/10/2019	1.417	50.688	Metabarcoding	Diatoms_counts		Phaeocystis_counts	PicoNano_counts	Crypto_counts
R1.20191031	R1	10	2019	31/10/2019	1.567	50.799	Metabarcoding	Diatoms_counts		Phaeocystis_counts	PicoNano_counts	Crypto_counts
R2.20191031	R2	10	2019	31/10/2019	1.542	50.799	Metabarcoding	Diatoms_counts	Dinoflagellates_counts	Phaeocystis_counts	PicoNano_counts	Crypto_counts
R4.20191031	R4	10	2019	31/10/2019	1.452	50.799	Metabarcoding					
S1.20191113	S1	11	2019	13/11/2019	1.521	50.688	Metabarcoding					
R1.20191121	R1	11	2019	21/11/2019	1.567	50.799	Metabarcoding					

R2.20191121	R2	11	2019	21/11/2019	1.542	50.799	Metabarcoding	Diatoms_counts	Dinoflagellates_counts	Phaeocystis_counts	PicoNano_counts	Crypto_counts
R4.20191121	R4	11	2019	21/11/2019	1.452	50.799	Metabarcoding	Diatoms_counts	Dinoflagellates_counts	Phaeocystis_counts	PicoNano_counts	Crypto_counts
R1.20191204	R1	12	2019	04/12/2019	1.567	50.799	Metabarcoding	Diatoms_counts		Phaeocystis_counts		
R2.20191204	R2	12	2019	04/12/2019	1.542	50.799	Metabarcoding	Diatoms_counts		Phaeocystis_counts		
R1.20191218	R1	12	2019	18/12/2019	1.567	50.799	Metabarcoding	Diatoms_counts		Phaeocystis_counts	PicoNano_counts	Crypto_counts
R4.20191218	R4	12	2019	18/12/2019	1.452	50.799		Diatoms_counts		Phaeocystis_counts		
R1.20200214	R1	2	2020	14/02/2020	1.567	50.799	Metabarcoding	Diatoms_counts	Dinoflagellates_counts	Phaeocystis_counts	PicoNano_counts	Crypto_counts
R4.20200214	R4	2	2020	14/02/2020	1.452	50.799	Metabarcoding	Diatoms_counts	Dinoflagellates_counts	Phaeocystis_counts	PicoNano_counts	Crypto_counts
S1.20200214	S1	2	2020	14/02/2020	1.521	50.688	Metabarcoding					
R1.20200520	R1	5	2020	20/05/2020	1.567	50.799	Metabarcoding	Diatoms_counts	Dinoflagellates_counts	Phaeocystis_counts	PicoNano_counts	Crypto_counts
R2.20200520	R2	5	2020	20/05/2020	1.542	50.799	Metabarcoding	Diatoms_counts	Dinoflagellates_counts	Phaeocystis_counts	PicoNano_counts	Crypto_counts
S1.20200525	S1	5	2020	25/05/2020	1.521	50.688	Metabarcoding	Diatoms_counts		Phaeocystis_counts		
R1.20200527	R1	5	2020	27/05/2020	1.567	50.799	Metabarcoding	Diatoms_counts	Dinoflagellates_counts	Phaeocystis_counts	PicoNano_counts	Crypto_counts
R2.20200527	R2	5	2020	27/05/2020	1.542	50.799		Diatoms_counts		Phaeocystis_counts	PicoNano_counts	Crypto_counts
R4.20200527	R4	5	2020	27/05/2020	1.452	50.799						
R1.20200528	R1	5	2020	28/05/2020	1.567	50.799	Metabarcoding	Diatoms_counts	Dinoflagellates_counts	Phaeocystis_counts	PicoNano_counts	Crypto_counts
R1.20200529	R1	5	2020	29/05/2020	1.567	50.799	Metabarcoding	Diatoms_counts	Dinoflagellates_counts	Phaeocystis_counts	PicoNano_counts	Crypto_counts
S1.20200603	S1	6	2020	03/06/2020	1.521	50.688	Metabarcoding	Diatoms_counts		Phaeocystis_counts	PicoNano_counts	Crypto_counts
S2.20200603	S2	6	2020	03/06/2020	1.417	50.688	Metabarcoding	Diatoms_counts	Dinoflagellates_counts	Phaeocystis_counts	PicoNano_counts	Crypto_counts
R1.20200604	R1	6	2020	04/06/2020	1.567	50.799		Diatoms_counts		Phaeocystis_counts	PicoNano_counts	Crypto_counts
R2.20200604	R2	6	2020	04/06/2020	1.542	50.799	Metabarcoding	Diatoms_counts	Dinoflagellates_counts	Phaeocystis_counts	PicoNano_counts	Crypto_counts
R1.20200609	R1	6	2020	09/06/2020	1.567	50.799	Metabarcoding	Diatoms_counts	Dinoflagellates_counts	Phaeocystis_counts	PicoNano_counts	Crypto_counts
R1.20200610	R1	6	2020	10/06/2020	1.567	50.799	Metabarcoding	Diatoms_counts	Dinoflagellates_counts	Phaeocystis_counts	PicoNano_counts	Crypto_counts
R2.20200610	R2	6	2020	10/06/2020	1.542	50.799	Metabarcoding	Diatoms_counts	Dinoflagellates_counts	Phaeocystis_counts	PicoNano_counts	Crypto_counts
R4.20200610	R4	6	2020	10/06/2020	1.452	50.799	Metabarcoding	Diatoms_counts	Dinoflagellates_counts	Phaeocystis_counts	PicoNano_counts	Crypto_counts
R1.20200612	R1	6	2020	12/06/2020	1.567	50.799	Metabarcoding	Diatoms_counts	Dinoflagellates_counts	Phaeocystis_counts	PicoNano_counts	Crypto_counts
R1.20200615	R1	6	2020	15/06/2020	1.567	50.799	Metabarcoding	Diatoms_counts		Phaeocystis_counts	PicoNano_counts	Crypto_counts
R4.20200615	R4	6	2020	15/06/2020	1.452	50.799	Metabarcoding	Diatoms_counts		Phaeocystis_counts	PicoNano_counts	
R1.20200616	R1	6	2020	16/06/2020	1.567	50.799	Metabarcoding	Diatoms_counts	Dinoflagellates_counts	Phaeocystis_counts	PicoNano_counts	Crypto_counts
R1.20200616	R1	6	2020	16/06/2020	1.567	50.799		Diatoms_counts	Dinoflagellates_counts	Phaeocystis_counts	PicoNano_counts	Crypto_counts
R4.20200616	R4	6	2020	16/06/2020	1.452	50.799	Metabarcoding					

R1.20200617	R1	6	2020	17/06/2020	1.567	50.799	Metabarcoding	Diatoms_counts		Phaeocystis_counts	PicoNano_counts	Crypto_counts
R2.20200617	R2	6	2020	17/06/2020	1.542	50.799	Metabarcoding	Diatoms_counts	Dinoflagellates_counts	Phaeocystis_counts	PicoNano_counts	Crypto_counts
R4.20200617	R4	6	2020	17/06/2020	1.452	50.799		Diatoms_counts		Phaeocystis_counts	PicoNano_counts	Crypto_counts
R1.20200618	R1	6	2020	18/06/2020	1.567	50.799		Diatoms_counts	Dinoflagellates_counts	Phaeocystis_counts	PicoNano_counts	Crypto_counts
R4.20200618	R4	6	2020	18/06/2020	1.452	50.799	Metabarcoding	Diatoms_counts		Phaeocystis_counts	PicoNano_counts	Crypto_counts
R1.20200619	R1	6	2020	19/06/2020	1.567	50.799	Metabarcoding	Diatoms_counts	Dinoflagellates_counts	Phaeocystis_counts	PicoNano_counts	Crypto_counts
S1.20200623	S1	6	2020	23/06/2020	1.521	50.688	Metabarcoding	Diatoms_counts		Phaeocystis_counts	PicoNano_counts	Crypto_counts
R1.20200624	R1	6	2020	24/06/2020	1.567	50.799	Metabarcoding	Diatoms_counts	Dinoflagellates_counts	Phaeocystis_counts	PicoNano_counts	Crypto_counts
R2.20200624	R2	6	2020	24/06/2020	1.542	50.799	Metabarcoding	Diatoms_counts	Dinoflagellates_counts	Phaeocystis_counts	PicoNano_counts	Crypto_counts
R1.20200625	R1	6	2020	25/06/2020	1.567	50.799	Metabarcoding	Diatoms_counts	Dinoflagellates_counts	Phaeocystis_counts	PicoNano_counts	Crypto_counts
S1.20200707	S1	7	2020	07/07/2020	1.521	50.688	Metabarcoding	Diatoms_counts		Phaeocystis_counts		
R1.20200715	R1	7	2020	15/07/2020	1.567	50.799	Metabarcoding					
R2.20200715	R2	7	2020	15/07/2020	1.542	50.799	Metabarcoding	Diatoms_counts		Phaeocystis_counts	PicoNano_counts	Crypto_counts
R4.20200715	R4	7	2020	15/07/2020	1.452	50.799	Metabarcoding	Diatoms_counts	Dinoflagellates_counts	Phaeocystis_counts	PicoNano_counts	Crypto_counts
R1.20200721	R1	7	2020	21/07/2020	1.567	50.799	Metabarcoding	Diatoms_counts	Dinoflagellates_counts	Phaeocystis_counts	PicoNano_counts	Crypto_counts
R2.20200721	R2	7	2020	21/07/2020	1.542	50.799	Metabarcoding	Diatoms_counts	Dinoflagellates_counts	Phaeocystis_counts	PicoNano_counts	Crypto_counts
R4.20200721	R4	7	2020	21/07/2020	1.452	50.799	Metabarcoding	Diatoms_counts	Dinoflagellates_counts	Phaeocystis_counts	PicoNano_counts	Crypto_counts
S1.20200722	S1	7	2020	22/07/2020	1.521	50.688	Metabarcoding	Diatoms_counts	Dinoflagellates_counts	Phaeocystis_counts	PicoNano_counts	Crypto_counts
S2.20200722	S2	7	2020	22/07/2020	1.417	50.688	Metabarcoding	Diatoms_counts	Dinoflagellates_counts	Phaeocystis_counts	PicoNano_counts	Crypto_counts
R1.20200730	R1	7	2020	30/07/2020	1.567	50.799	Metabarcoding	Diatoms_counts	Dinoflagellates_counts	Phaeocystis_counts	PicoNano_counts	Crypto_counts
R1.20200730	R1	7	2020	30/07/2020	1.567	50.799		Diatoms_counts	Dinoflagellates_counts	Phaeocystis_counts	PicoNano_counts	Crypto_counts
R2.20200730	R2	7	2020	30/07/2020	1.542	50.799	Metabarcoding	Diatoms_counts	Dinoflagellates_counts	Phaeocystis_counts	PicoNano_counts	Crypto_counts
R4.20200730	R4	7	2020	30/07/2020	1.452	50.799	Metabarcoding	Diatoms_counts		Phaeocystis_counts		
R1.20200901	R1	9	2020	01/09/2020	1.567	50.799	Metabarcoding					
R4.20200901	R4	9	2020	01/09/2020	1.452	50.799	Metabarcoding					
S1.20200902	S1	9	2020	02/09/2020	1.521	50.688	Metabarcoding					
S2.20200902	S2	9	2020	02/09/2020	1.417	50.688	Metabarcoding					
R1.20200904	R1	9	2020	04/09/2020	1.567	50.799	Metabarcoding					
R4.20200904	R4	9	2020	04/09/2020	1.452	50.799	Metabarcoding					
R1.20200907	R1	9	2020	07/09/2020	1.567	50.799	Metabarcoding					
R4.20200907	R4	9	2020	07/09/2020	1.452	50.799	Metabarcoding					

R1.20200911	R1	9	2020	11/09/2020	1.567	50.799	Metabarcoding					
R2.20200911	R2	9	2020	11/09/2020	1.542	50.799	Metabarcoding					
R4.20200911	R4	9	2020	11/09/2020	1.452	50.799	Metabarcoding					
R4.20200914	R4	9	2020	14/09/2020	1.452	50.799	Metabarcoding					
S1.20200916	S1	9	2020	16/09/2020	1.521	50.688	Metabarcoding	Diatoms_counts		Phaeocystis_counts	PicoNano_counts	Crypto_counts
S2.20200916	S2	9	2020	16/09/2020	1.417	50.688	Metabarcoding					
R1.20200917	R1	9	2020	17/09/2020	1.567	50.799	Metabarcoding					
R2.20200917	R2	9	2020	17/09/2020	1.542	50.799	Metabarcoding					
R1.20200929	R1	9	2020	29/09/2020	1.567	50.799	Metabarcoding					
R2.20200929	R2	9	2020	29/09/2020	1.542	50.799	Metabarcoding					
R4.20200929	R4	9	2020	29/09/2020	1.452	50.799	Metabarcoding					
R1.20200930	R1	9	2020	30/09/2020	1.567	50.799	Metabarcoding					
R4.20200930	R4	9	2020	30/09/2020	1.452	50.799	Metabarcoding					
S1.20201001	S1	10	2020	01/10/2020	1.521	50.688	Metabarcoding	Diatoms_counts	Dinoflagellates_counts	Phaeocystis_counts	PicoNano_counts	Crypto_counts
S2.20201001	S2	10	2020	01/10/2020	1.417	50.688	Metabarcoding					
R1.20201002	R1	10	2020	02/10/2020	1.567	50.799	Metabarcoding					
R1.20201009	R1	10	2020	09/10/2020	1.567	50.799	Metabarcoding					
R2.20201009	R2	10	2020	09/10/2020	1.542	50.799	Metabarcoding					
R4.20201009	R4	10	2020	09/10/2020	1.452	50.799	Metabarcoding					
S1.20201015	S1	10	2020	15/10/2020	1.521	50.688	Metabarcoding	Diatoms_counts		Phaeocystis_counts	PicoNano_counts	Crypto_counts
S2.20201015	S2	10	2020	15/10/2020	1.417	50.688	Metabarcoding					
R1.20201016	R1	10	2020	16/10/2020	1.567	50.799	Metabarcoding					
R2.20201016	R2	10	2020	16/10/2020	1.542	50.799	Metabarcoding					
R4.20201016	R4	10	2020	16/10/2020	1.452	50.799	Metabarcoding					
**Number of samples**							**287**	**251**	**154**	**251**	**233**	**231**

Table S3. Cell to carbon biomass conversion factors used in this study for the estimation of biomass of the different phytoplankton groups identified in the eastern English Channel at the SOMLIT and DYPHYRAD stations from March 2016 to October 2020.

	Conversion factor	References
Diatoms	pgC $cell^{-1}$=0.288 x $(biovolume)^{0.811}$	Based on linear dimensions the biovolume was calculated according to the cell shape (Hillebrand et al 1999). Conversion from biovolume to biomass according to Menden Deuer & Lessard 2000 and according to microscopic observations over the period 2007-2015 (Breton et al., 2017)
Dinoflagelates	pgC $cell^{-1}$=0.76 x $(biovolume)^{0.819}$	Based on linear dimensions the biovolume was calculated according to the cell shape (Hillebrand et al 1999). Conversion from biovolume to biomass according to Menden Deuer & Lessard 2000 and according to microscopic observations over the period 2018-2020 (this study)
Phaeocystis free flagellate cell	8 pgC $cell^{-1}$	Schoemann et al., 2005
Phaeocystis colonial cells	14.2 pgC $cell^{-1}$	
Nanophytoplankton	4.98 pgC $cell^{-1}$	Cell carbon was estimated using the empirical relationship between biovolume and cell carbon of Verity et al. (1992). 0.433x $(biovolume)^{0.86}$.Mean ESD=3.03 µm (ecquivalent sphere diameter) according to microscopic observations
Picophytoplankton	1.19 pgC $cell^{-1}$	As above, based on mean ESD=1.74
Cryptophytes	11 pgC $cell^{-1}$	Conversion from biovolume to biomass according to Menden Deuer & Lessard 2000 and according to microscopic observations over the period 2007-2015 (Breton et al., 2017)

Table S4. Monthly range, mean (±SD) and median values of the mean Photosynthetic Active Radiation (PAR_{10m}, E m^{-2} d^{-1}), sea surface temperature (T, °C), salinity (S, PSU), nutrients (nitrite and nitrate: NO_2 + NO_3, phosphate: PO_4, all in µM, the N/P molar ratio, silicate $Si(OH)_4$, µM), chlorophyll-a (Chl-a, µg L^{-1}), rainfall (Kg m^2), and wind stress (Pa) in the eastern English Channel at the SOMLIT (S1, S2) and DYPHYRAD (R1, R2, R4) stations from March 2016 to October 2020. Note that only three samples were available for August (with grey color).

	January	February	March	April	May	June	July	August	September	October	November	December
PAR_{10m} (E m^{-2} d^{-1})												
Range	17.4-21.3	23.2-35.5	36.2-52.8	56.5-69.3	73.03-83.86	84.3-86.7	79.7-86.7	66.5-66.5	46.6-64.0	29.4-46.4	20.2-28.3	14.8-17.5
Mean±SD	19.2±1.6	29.1±3.8	46.7±4.7	62.3±3.1	79.67±3.57	86.1±0.7	84.2±2.1	66.5±0.0	55.6±5.7	39.7±5.5	22.7±2.8	16.0±1.0
Median	19	28.6	48.2	63.7	81.27	86.4	84.9	66.5	55.2	38.3	22.2	15.9
CV(%)	8.4	13.1	10.0	5.1	4.48	0.9	2.5	0.0	10.2	14.2	12.5	6
T (°C)												
Range	5.4-7.9	6.0-8.3	5.8-9.5	8.6-11.4	11.03-14.7	13.7-18.2	16.5-20.1	19.1-19.5	12.4-19.8	14.4-18.1	10.6-14.9	8.1-11.4
Mean±SD	7.1±0.8	7.4±0.7	8.0±1.3	9.6±0.7	12.91±1.06	15.4±1.0	17.8±0.8	19.3±0.2	18.1±1.3	16.2±0.9	12.6±1.3	10.2±0.8
Median	7	7.5	8.2	9.7	12.86	15.4	17.8	19.5	18.6	16.4	12.8	10.4
CV(%)	11.8	10.2	16.6	7.5	8.19	6.4	4.2	1.2	7.4	5.4	10.4	7.9
S (µM)												
Range	33.8-34.9	32.9-34.8	33.3-34.9	32.6-35.2	33.5-35.0	31.2-35.1	33.4-34.9	0.03-0.1	33.8-34.9	33.6-35.1	34.1-35.0	33.9-35.0
Mean±SD	34.2±0.4	34.0±0.6	34.2±0.5	34.1±0.6	34.2±0.4	34.3±0.5	34.6±0.3	34.6±0.2	34.5±0.2	34.5±0.3	34.6±0.3	34.4±0.3
Median	34.1	34.1	34.2	34.2	34.09	34.4	34.6	34.6	34.6	34.5	34.6	34.3
CV(%)	1.0	1.7	1.5	1.7	1.27	1.4	0.9	0.5	0.5	0.8	0.8	0.8
NO_2+NO_3 (µM)												
Range	3.4-10.6	0.3-23.9	1.1-20.7	0.3-9.3	0.14-1.6	0.01-4.4	0.2-1.1	0.2-0.5	0.3-2.8	0.2-3.5	0.4-5.2	1.1-8.6
Mean±SD	8.0±4.0	7.6±6.6	5.2±4.4	2.2±2.3	0.53±0.4	0.9±1.0	0.5±0.2	0.4±0.2	1.0±0.6	1.5±0.8	2.2±1.4	3.5±2.4
Median	10.0	4.2	4.2	1.5	0.41	0.5	0.5	0.5	0.7	1.5	1.8	2.9
CV(%)	49.8	86.5	85.2	104.1	74.01	113.5	42.0	44.2	67.8	52.4	66.3	68.6
PO_4 (µM)												
Range	0.4-0.6	0.2-1.0	0.01-0.6	0.03-0.3	0.01-0.29	0.01-0.4	0.01-0.2	0.1-0.2	0.01-0.5	0.03-0.4	0.01-0.5	0.1-0.8
Mean±SD	0.5±0.1	0.5±0.2	0.3±0.1	0.1±0.1	0.1±0.07	0.1±0.1	0.1±0.05	0.1±0.1	0.17±0.1	0.2±0.1	0.3±0.2	0.4±0.2
Median	0.5	0.5	0.2	0.2	0.11	0.1	0.1	0.1	0.14	0.2	0.2	0.4
CV(%)	14.0	39.3	51.3	47	71.24	91.7	57.0	52.2	64.0	46.5	56.4	47.3
N/P												
Range	7.0-24.2	0.4-35.2	4.8-52.7	1.5-43.7	0.82-262.5	2.1-316.1	1.1-15.3	3.0-3.9	2.3-78.0	2.7-16.3	0.4-5.2	3.8-21.1
Mean±SD	16.6±8.7	14.7±10.5	18.5±13.2	13.0±12.0	25.71±61.0	17.4±42.4	7.3±3.3	3.4±0.4	8.4±12.2	7.7±2.7	2.2±1.4	9.1±4.6
Median	18.5	9.4	14.6	9.3	4.22	7.5	6.5	3.3	5.3	7.1	1.8	9.0
CV(%)	52.8	71.8	71.7	92.0	237.3	243.6	44.8	12.2	145.0	35.0	66.3	50.2
$Si(OH)_4$ (µM)												
Range	4.6-8.8	1.8-14.1	0.1-5.2	0.1-3.6	0.05-2.68	0.01-3.0	0.01-3.4	1.1-2.9	0.01-4.8	0.1-6.1	0.3-5.5	1.0-9.3
Mean±SD	5.9±1.9	5.2±3.6	1.9±1.5	0.9±0.8	1.04±0.70	0.9±0.9	1.3±1.1	2.1±0.9	1.2±1.1	1.8±1.7	2.7±1.5	4.6±2.7
Median	5.1	3.3	1.8	0.6	0.9	0.6	1.2	2.3	0.7	1.5	3.1	4.3
CV(%)	33	68.3	79.2	88.9	67.52	96.5	84.2	41.9	92.8	92.7	53.5	58.6
Chl-a (µg L^{-1})												
Range	1.01-2.77	0.6-7.9	1.6-9.3	0.6-15.2	0.6-10.9	0.6-9.6	0.6-8.5	0.8-1.5	0.5-7.9	1.0-6.9	0.7-4.6	0.5-3.5
Mean±SD	1.7±0.6	3.9±2.2	5.1±2.3	5.7±3.1	2.9±3.0	2.3±1.7	2.1-1.5	1.1±0.3	1.6±1.2	2.4±1.3	2.5±1.4	1.5±0.9
Median	1.6	3.5	5.1	5.4	1.7	1.8	1.7	0.9	1.3	2.0	2.6	1.3
CV(%)	33.7	56.7	44.3	54.9	102.2	74.1	70.8	32.6	73.5	53.0	56.9	61.2
Rainfall (Kg m^2)												
Range	1.0-2.6	0.0002-5.5	0.1-4.3	0.01-3.2	0.02-4.6	0.02-4.6	0.01-3.8	2.3-2.4	0.05-6.5	0.1-8.0	0.2-4.6	0.05-0.1
Mean±SD	1.8±0.6	1.8±2.1	1.6±1.4	1.6±1.2	1.3±1.4	1.3±1.4	1.0±1.1	2.4±0.0	2.7±2.4	2.7±2.1	2.0-1.7	0.1±0.03
Median	1.7	1.0	1.1	2.5	0.7	0.7	0.6	2.4	2.4	2.4	1.4	0.1
CV(%)	36	117.9	88.2	75.5	103.2	103.2	109.5	0.5	87	77.8	83.3	32.1
Wind stress (Pa)												
Range	0.03-0.1	0.05-0.2	0.02-0.2	0.02-0.1	0.01-0.07	0.01-0.1	0.01-0.1	0.1-0.1	0.02-0.2	0.03-0.1	0.02-0.1	0.05-0.1
Mean±SD	0.1±0.03	0.1±0.1	0.1±0.1	0.04±0.02	0.04±0.02	0.04±0.02	0.04±0.02	0.1±0.0	0.06±0.04	0.1±0.04	0.1±0.03	0.1±0.03
Median	0.05	0.1	0.1	0.04	0.04	0.04	0.03	0.1	0.04	0.1	0.1	0.1
CV(%)	52.6	60.3	69.6	41.6	43.45	42.7	61.1	2.5	70.3	49.2	42.9	32.1

*Table S5. Permutation test for distance-based redundancy analysis (db-RDA) under the reduced model of phytoplankton communities based on metabarcoding data in relation to environmental variables in the eastern English Channel at the SOMLIT and DYPHYRAD stations from March 2016 to October 2020. P-value significant codes: 0 '***', 0.001 '**', 0.01 '*', 0.05 '.', 0.1, ' ' 1.*

Parameter	Unit	df	Sum of squares	Pseudo-F	Pseudo-r^2	p-value
PAR_{10m}	$E\ m^{-2}\ d^{-1}$	1	6.49	21.53	0.001	***
SST	^{o}C	1	5.43	18.05	0.001	***
SSU	nu	1	0.75	2.49	0.002	**
NO2+NO3	µM	1	1.39	4.62	0.001	***
PO4	µM	1	0.91	3.03	0.001	***
SiO4	µM	1	0.75	2.49	0.001	***
Chla	$mg\ L^{-1}$	1	0.81	2.68	0.001	***
Rainfall	$Kg\ m^{2}$	1	0.52	1.73	0.02	*
Wind Stress	Pa	1	0.49	1.62	0.029	*
Residual	235	70.65				

*Table S6. Permutation test for distance-based redundancy analysis (db-RDA) under the reduced model of phytoplankton communities based on morphological data in relation to environmental variables in the eastern English Channel at the SOMLIT and DYPHYRAD stations from March 2016 to October 2020. P-value significant codes: 0 '***', 0.001 '**', 0.01 '*', 0.05 '.', 0.1 ' ', 1.*

Parameter	Unit	df	Sum of squares	Pseudo-F	Pseudo-r^2	p-value
PAR_{10m}	$E\ m^{-2}\ d^{-1}$	1	7.47	23.50	0.001	***
T	^{o}C	1	3.14	9.88	0.001	***
S	nu	1	0.40	1.25	0.210	n.s.
NO_2+NO_3	µM	1	0.94	2.95	0.001	***
PO_4	µM	1	1.40	4.41	0.001	***
$Si(OH)_4$	µM	1	0.76	2.10	0.013	*
Chl-a	$\mu g\ L^{-1}$	1	1.63	5.12	0.001	***
Rainfall	$Kg\ m^{2}$	1	0.86	2.71	0.003	**
Wind Stress	Pa	1	0.57	1.79	0.035	*
Residual		212	67.38			

Table S7: The relative contribution of each ecological process in community assembly is presented by the percentage per month (see also Fig. 6).

Month	Heterogeneous selection	**Homogeneous selection**	Dispersal limitation	Homogeneous dispersal	**Drift**
January	0	**83**	17	0	0
Fabruary	0	**52**	8	19	19
March	0	**54**	4	11	31
April	1	8	11	11	**69**
May	0	2	6	6	**87**
June	0	3	16	10	**72**
July	1	12	8	4	**75**
August	0	0	33	33	33
September	2	**38**	4	4	**52**
October	0	**30**	2	8	**60**
November	0	**28**	1	7	**65**
December	0	**25**	4	10	**61**

*Table S8. Permutational multivariate analysis of variance (PERMANOVA) between the phylogenetic community structure of eukaryotic phytoplankton (NRI) and the environmental variables measured in the eastern English Channel at the SOMLIT and DYPHYRAD stations from March 2016 to October 2020. P-value significant codes: 0 '***', 0.001 '**', 0.01 '*', 0.05 '.' ,0.1, ' ' 1.*

**Phylogenetic structure (NRI)**						
Parameter	Unit	df	Sum of squares	R^2	F	p-value
PAR_{10m}	$E\ m^{-2}\ d^{-1}$	1	0.69	0.02	7.76	***
T	^{o}C	1	2.48	0.07	27.95	***
S	nu	1	0.17	0.006	1.96	
NO_2+NO_3	µM	1	0.12	0.004	1.36	
PO_4	µM	1	0.15	0.005	1.67	
$Si(OH)_4$	µM	1	0.05	0.001	0.51	
Rainfall	$Kg\ m^2$	1	0.14	0.004	1.53	
Wind Stress	Pa	1	0.005	0.005	1.77	
Residual		236	21.98	0.670		
Total		244	31.13	1.00		

*Table S9. Permutational multivariate analysis of variance (PERMANOVA) between the phylogenetic community turnover of eukaryotic phytoplankton (betaNRI) and environmental variables measured in the eastern English Channel at the SOMLIT and DYPHYRAD stations from March 2016 to October 2020. P-value significant codes: 0 '***', 0.001 '**', 0.01 '*', 0.05 '.' ,0.1, ' ' 1.*

**Phylogenetic turnover (betaNRI)**						
Parameter	Unit	df	Sum of squares	R^2	F	p-value
PAR_{10m}	$E\ m^{-2}\ d^{-1}$	1	1.60	0.05	13.05	***
T	°C	1	2.27	0.07	18.55	***
S	nu	1	0.07	0.002	0.63	
NO_2+NO_3	µM	1	0.02	0.001	0.14	
PO_4	µM	1	0.06	0.002	0.52	
$Si(OH)_4$	µM	1	0.05	0.001	0.37	
Rainfall	$Kg\ m^2$	1	0.19	0.006	1.59	
Wind Stress	Pa	1	0.10	0.003	0.79	
Residual		236	28.85	0.87		
Total		244	33.58	1.00		

Supplementary Figures

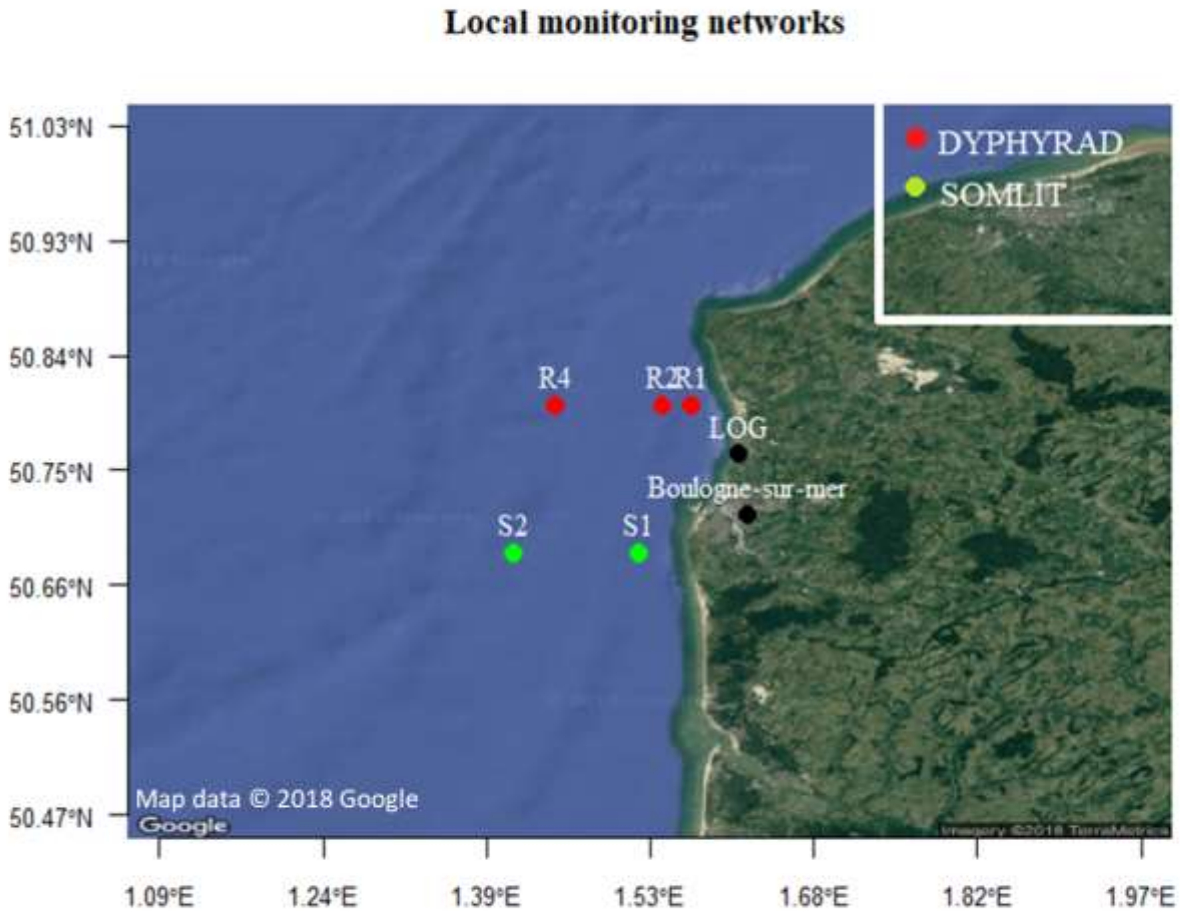

Figure S1. Location of the SOMLIT (S1, S2) and DYPHYRAD (R1, R2, R4) stations in the eastern English Channel (map creation with R software using the package Googlemap, Map data © 2018 Google).

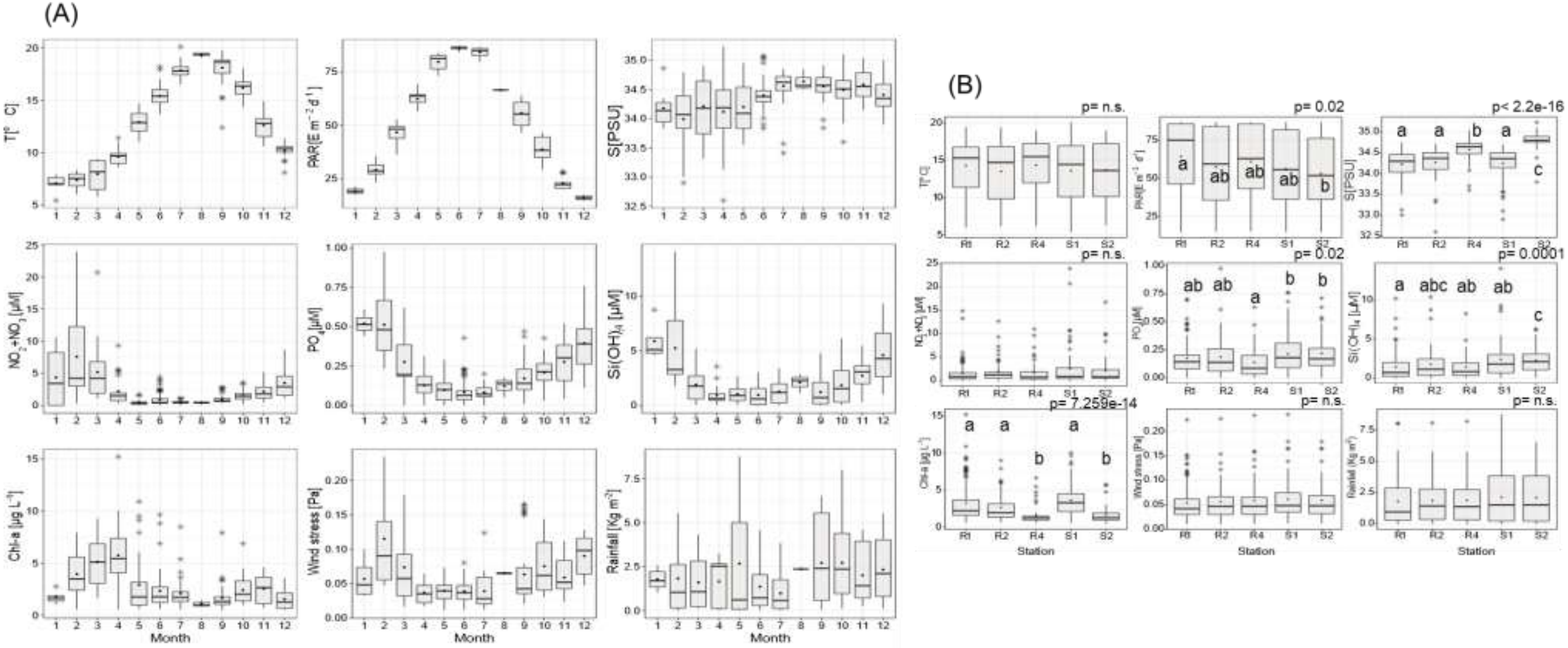

Figure S2. (A) Monthly variations of the environmental variables: Temperature (T, °C), Photosynthetic Active Radiation (PAR, E m^{-2} d^{-1}), Salinity (S, PSU), nitrite and nitrate (NO_2+NO_3 μM), phosphate (PO_4, μM), silicate ($Si(OH)_4$, μM), chlorophyll-a (Chl-a, μg L^{-1}), wind stress (Pa), rainfall (Kg m^{-2}) (B) Spatial variations of the environmental variables measured in the eastern English Channel at the DYPHYRAD and SOMLIT stations from March 2016 to October 2020. The letters indicate significant differences ($p < 0.05$) between stations based on Kruskal-Wallis and Nemenyi post-hoc test on the top of the graphs. Solid black lines represent the median, black dots the mean and the black stars the outliers.

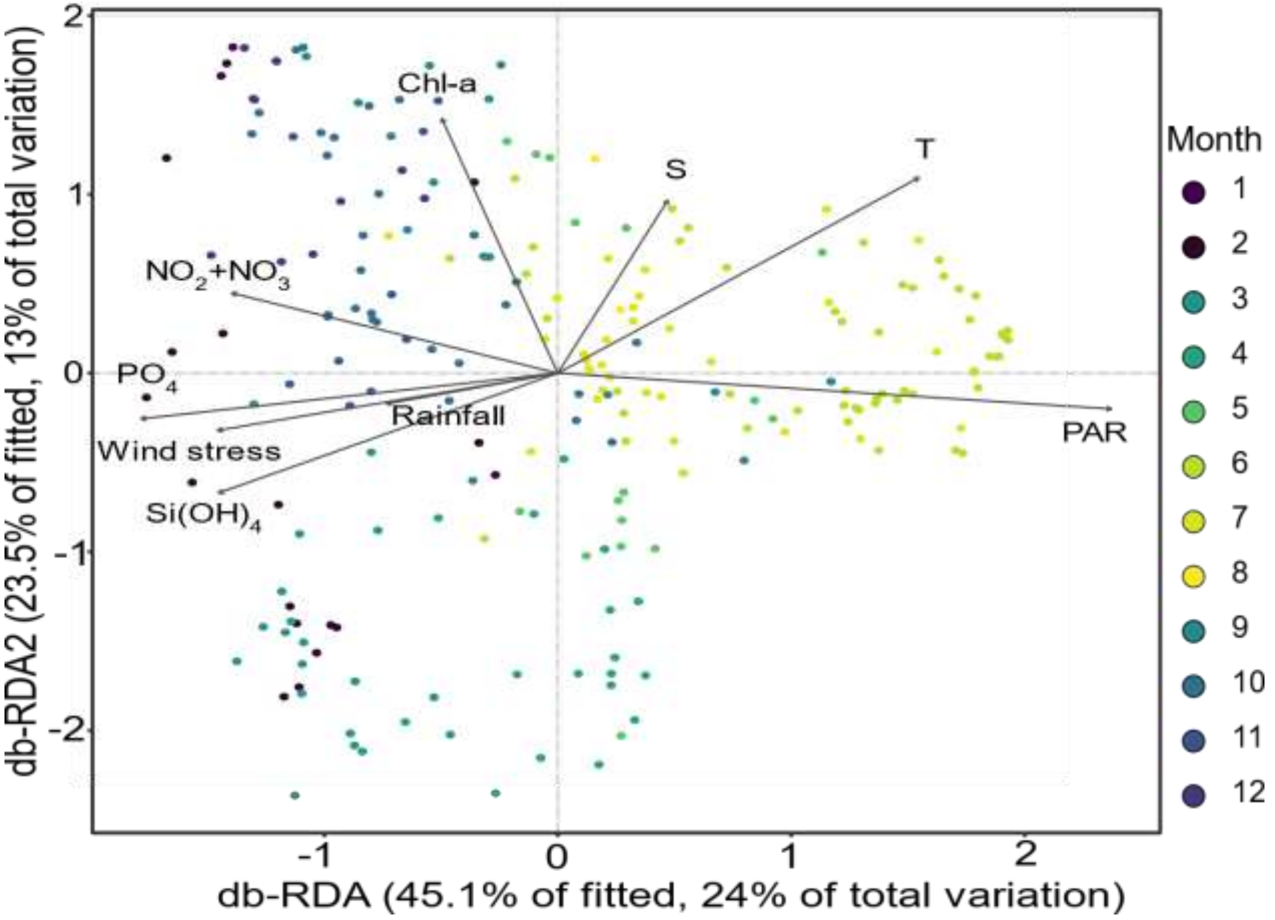

Figure S3. Distance-based redundancy (db-RDA) ordination plot of the phytoplankton communities based on morphological data (coloured dots with colour varying according to the months) to explore the link between the structure of the phytoplankton communities and the environmental variables (black arrows; temperature (T, °C), Photosynthetic Active Radiation (PAR, E m^{-2} d^{-1}), salinity (S, PSU), nitrite and nitrate (NO_2+NO_3 µM), phosphate (PO_4, µM), silicate ($Si(OH)_4$, µM), chlorophyll-a (Chl-a, µg L^{-1}), wind stress (Pa), rainfall (Kg m^{-2}) measured in the eastern English Channel at the DYPHYRAD and SOMLIT stations from March 2016 to October 2020.

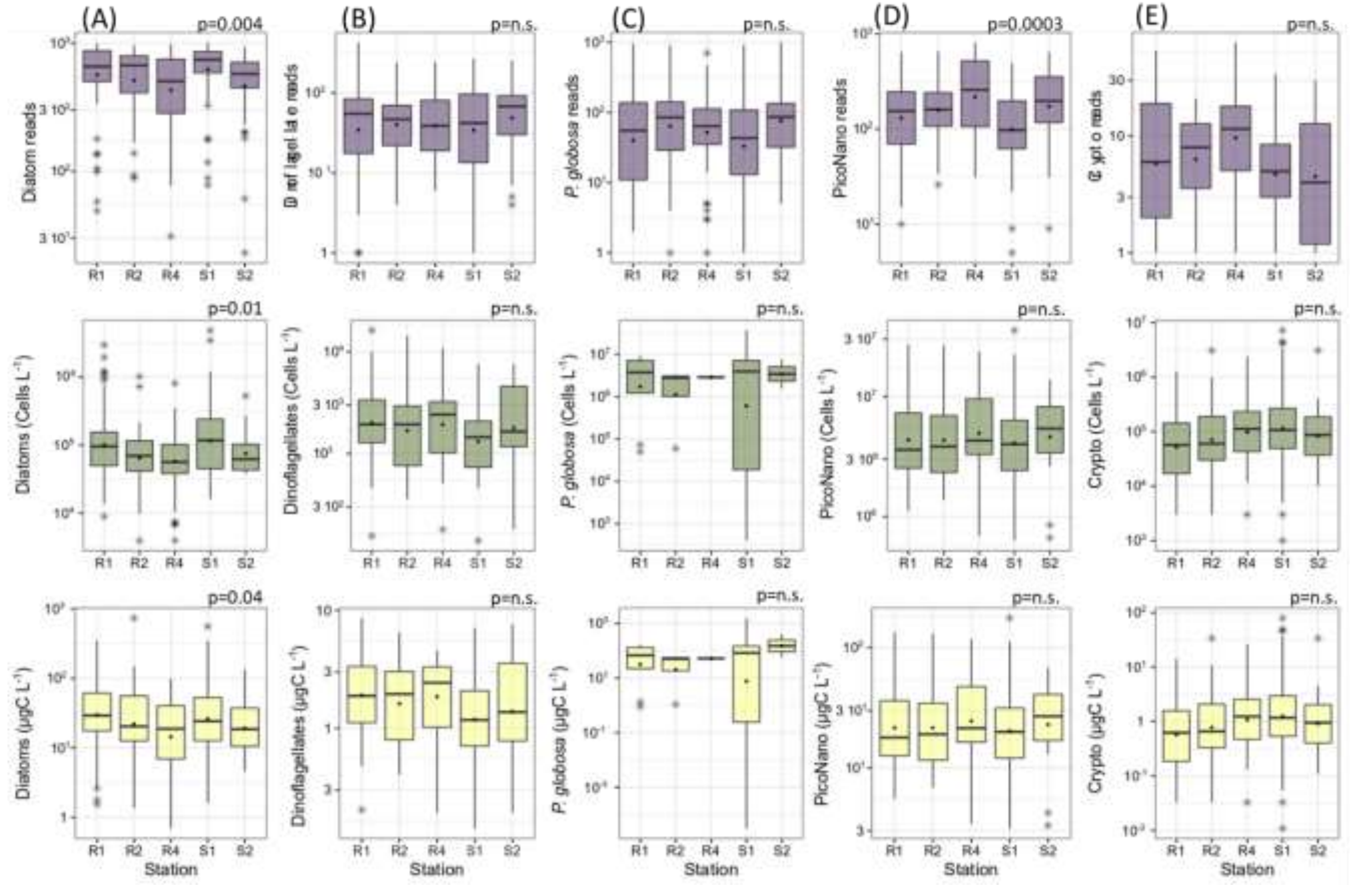

Figure S4. Spatial variations of the number of reads (purple), abundance (number of cell cell L^{-1}, in green) and biomass (µgC L^{-1}, in yellow) of the different phytoplankton groups (A: diatoms, B: dinoflagellates, C: P. globosa, D: pico- nanophytoplankton (PicoNano), and E: cryptophytes) identified in the eastern English Channel from March 2016 to October 2020. The letters indicate significant differences ($p < 0.05$) between stations based on Kruskal-Wallis and Nemenyi post-hoc test. Solid black lines represent the median, black dots the mean and the black stars the outliers.

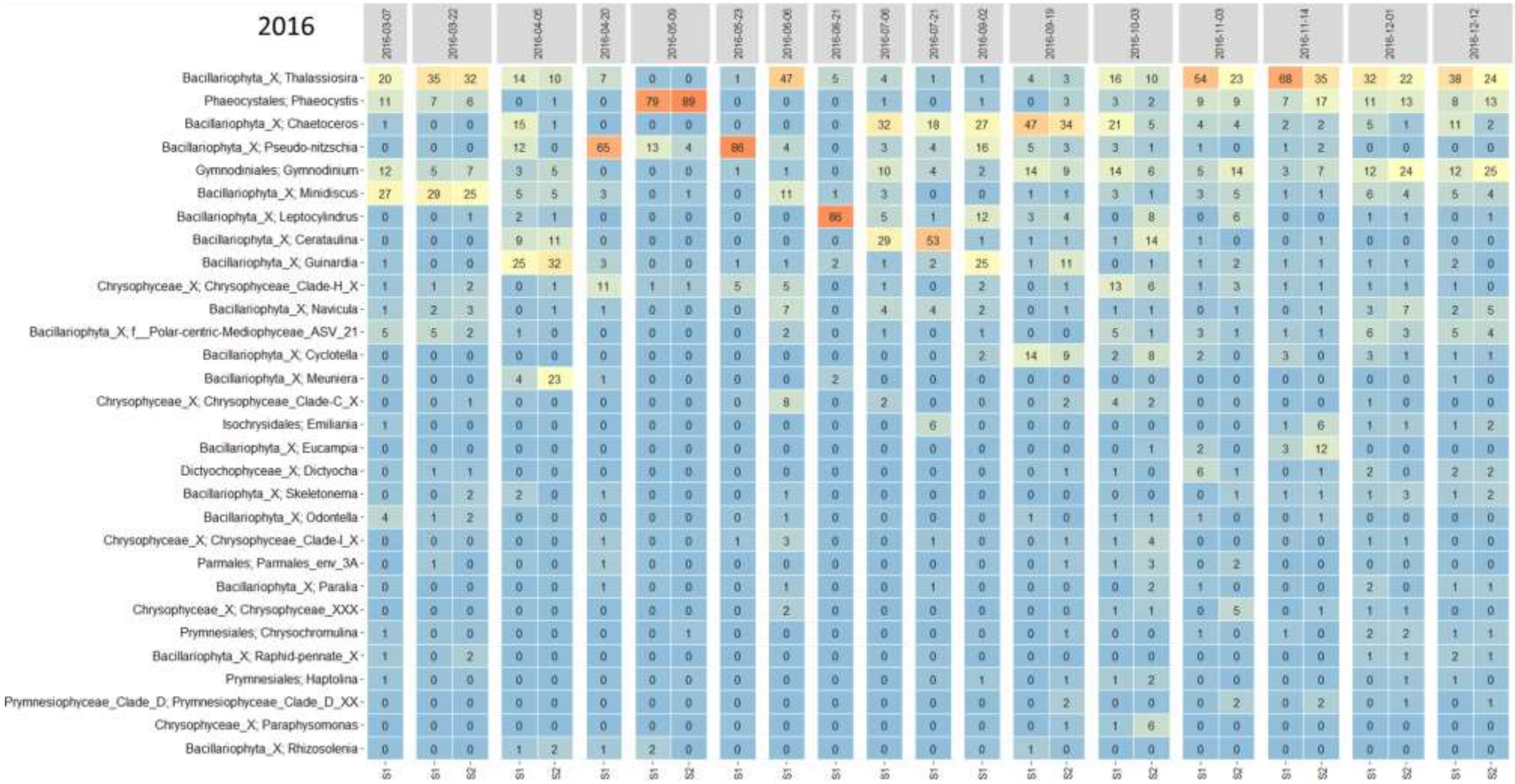

Figure S5. Heatmap illustrating the relative abundance of reads of the 30 most abundant phytoplankton genera (i.e., contributing to at least 0.5 % of reads in the whole data set), occurring in the eastern English Channel at the SOMLIT (S1, S2) stations in 2016.

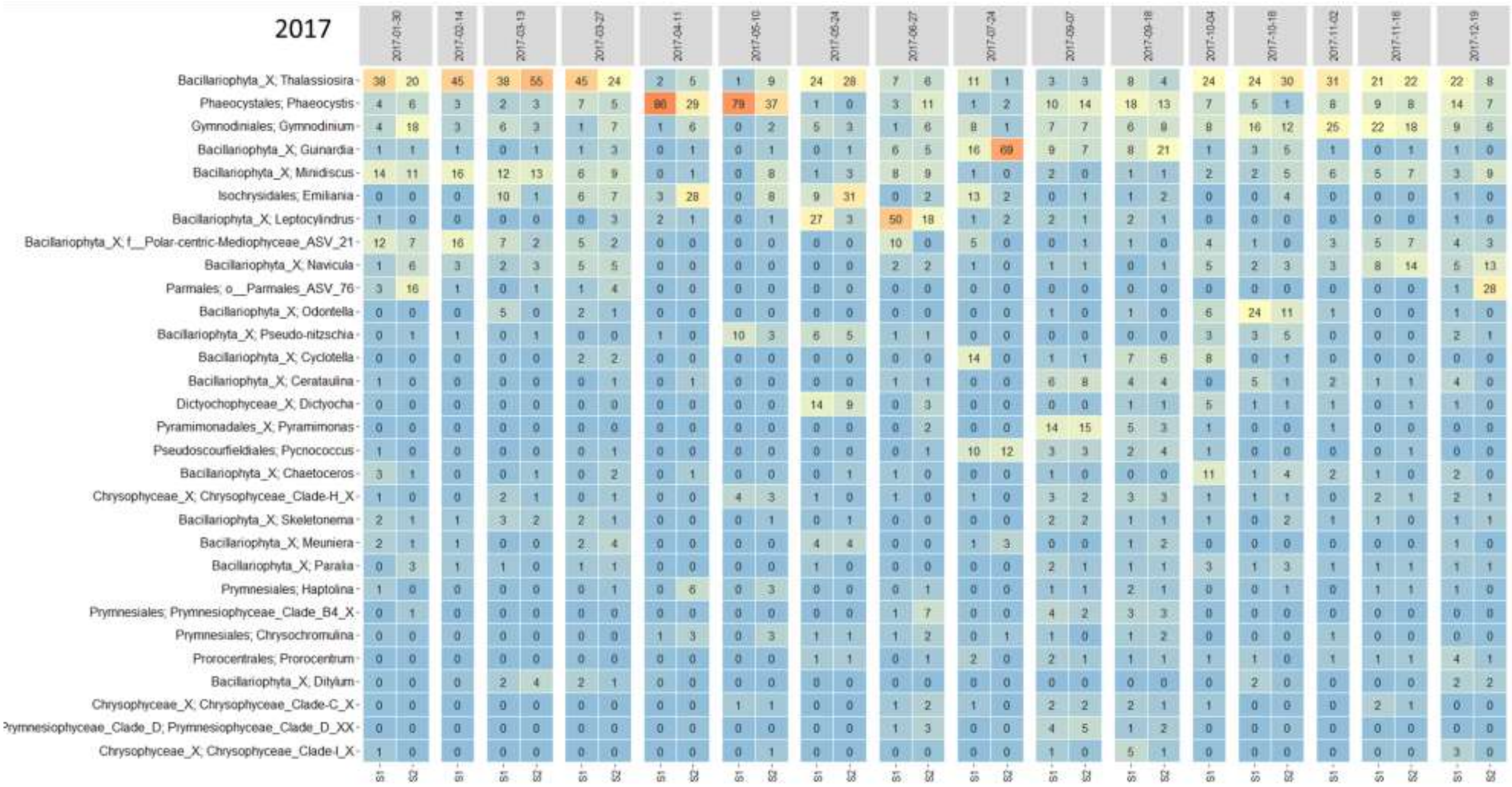

Figure S6. Heatmap illustrating the relative abundance of reads of the 30 most abundant phytoplankton genera (i.e., contributing to at least 0.5 % of reads in the whole data set), occurring in the eastern English Channel at the SOMLIT (S1, S2) stations in 2017.

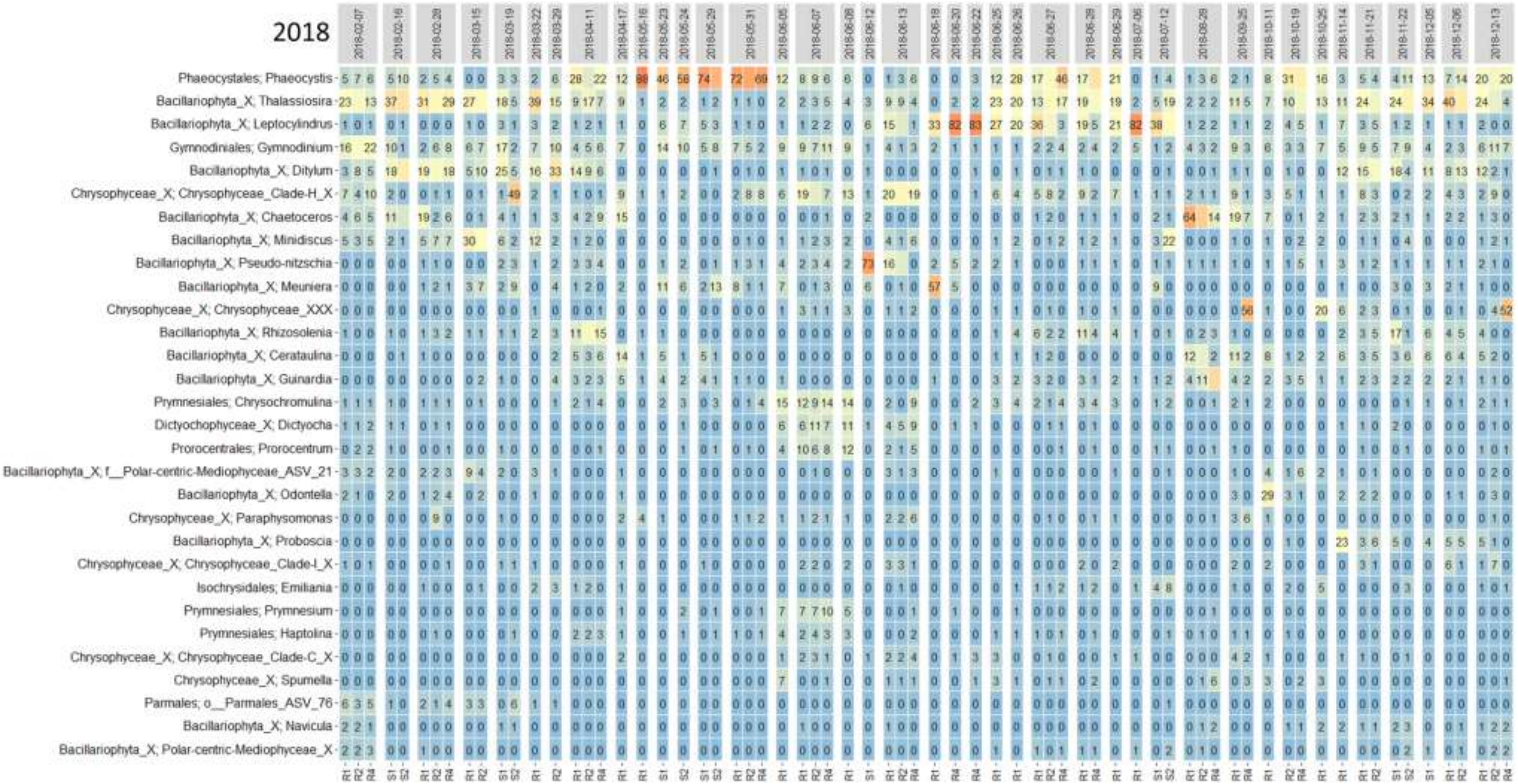

Figure S7. Heatmap illustrating the relative abundance of reads of the 30 most abundant phytoplankton genera (i.e., contributing to at least 0.5 % of reads in the whole data set), occurring in the eastern English Channel at the SOMLIT (S1, S2) and DYPHYRAD (R1, R2, R4) stations in 2018.

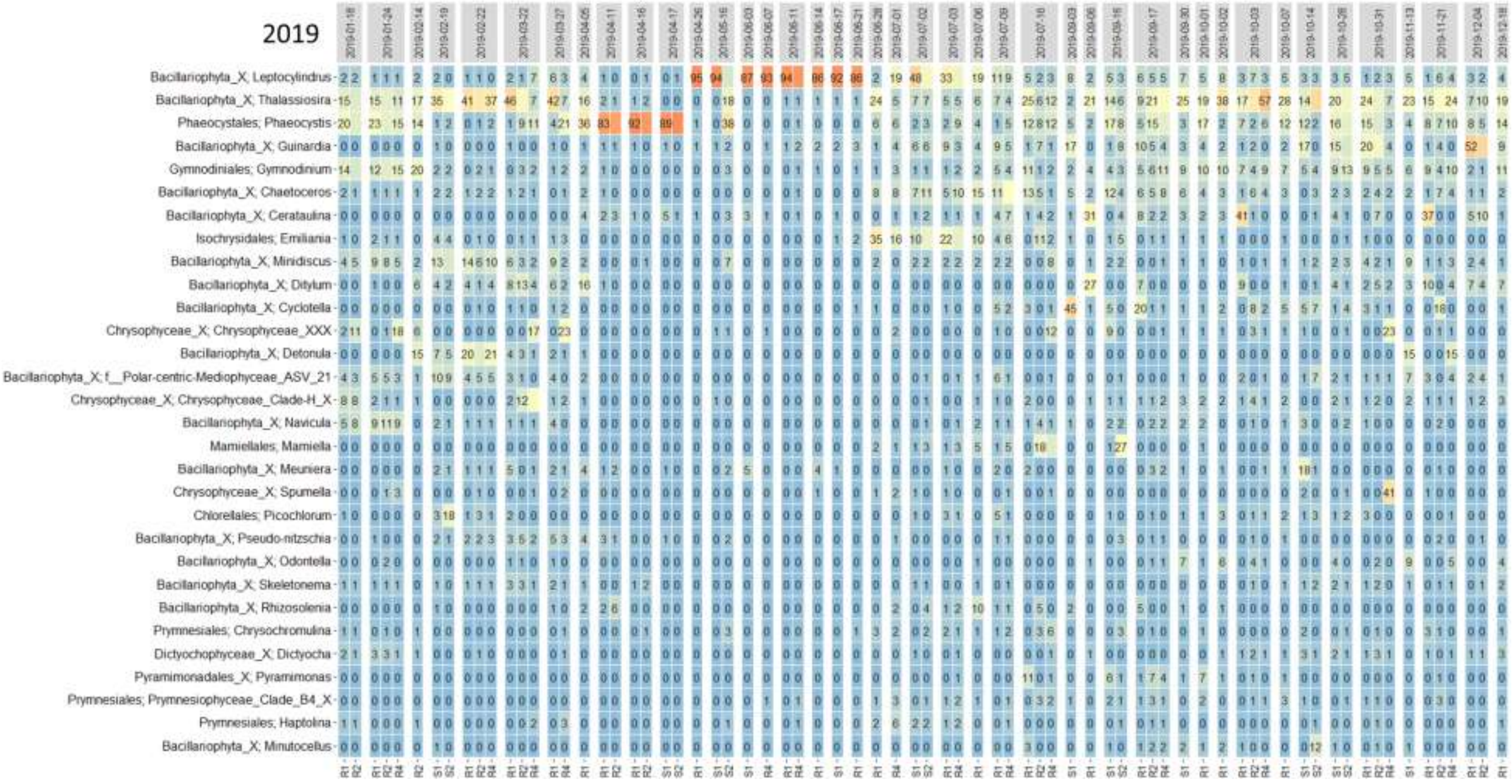

Figure S8. Heatmap illustrating the relative abundance of reads of the 30 most abundant phytoplankton genera (i.e., contributing to at least 0.5 % of reads in the whole data set), occurring in the eastern English Channel at the SOMLIT (S1, S2) and DYPHYRAD (R1, R2, R4) stations in 2019.

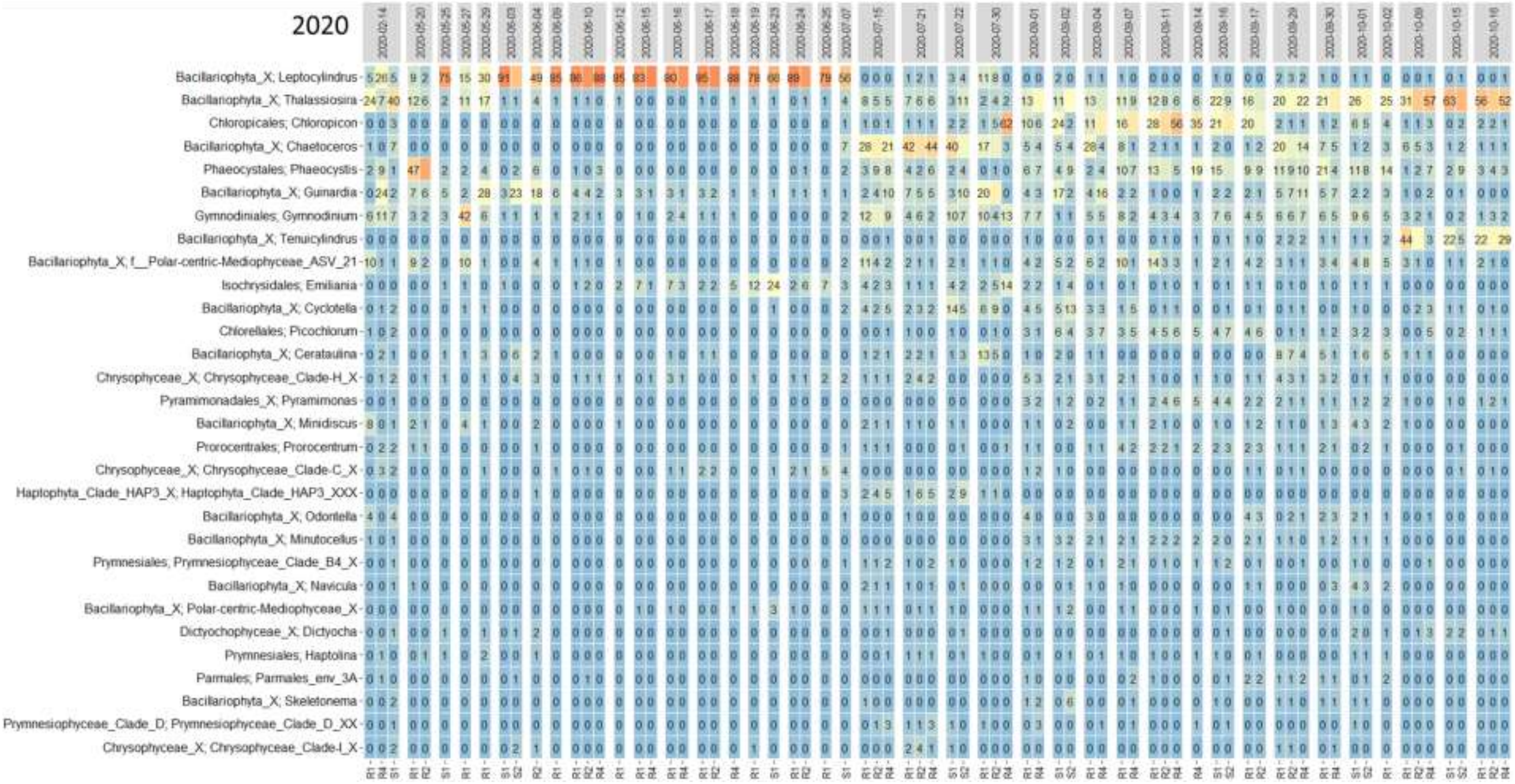

Figure S9. Heatmap illustrating the relative abundance of reads of the 30 most abundant phytoplankton genera (i.e., contributing to at least 0.5 % of reads in the whole data set), occurring in the eastern English Channel at the SOMLIT (S1, S2) and DYPHYRAD (R1, R2, R4) stations in 2020.

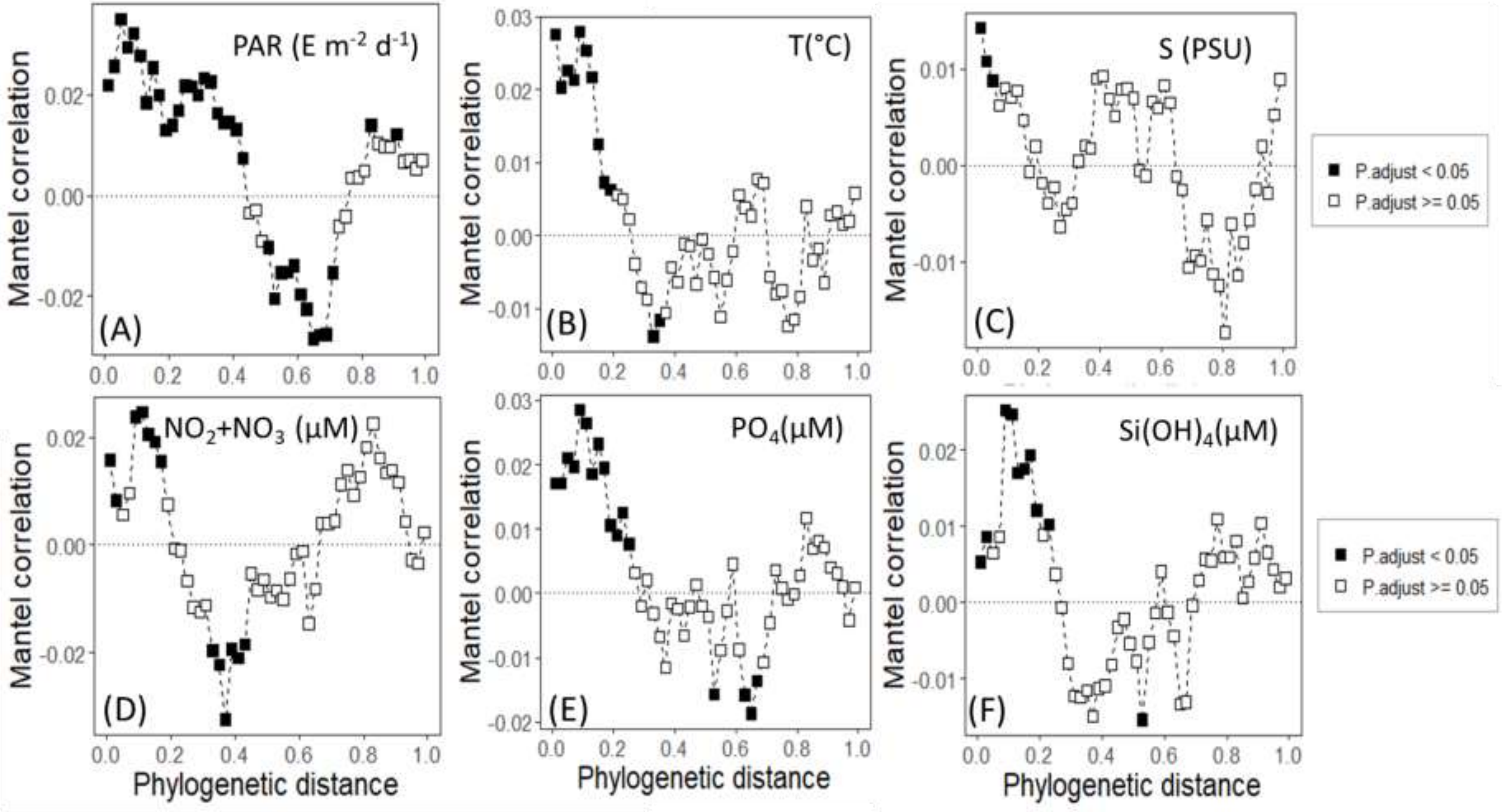

Figure S10. Exploration of the phylogenetic signal of the phytoplankton communities in the eastern English Channel at the SOMLIT and DYPHYRAD stations from March 2016 to October 2020 through Mantel correlograms between the Euclidean distance matrix of ASVs environmental optima for (A) Photosynthetic Active Radiation (PAR, E m^{-2} d^{-1}), (B) temperature (T, °C), (C) salinity (S, PSU), (D) nitrite and nitrate (NO_2+NO_3 µM), (E) phosphate (PO_4, µM), (F) silicate ($Si(OH)_4$, µM), and the phylogenetic distance matrix. Black and white squares indicate significant ($p<0.05$) and non-significant correlation values, respectivel
